# DNA methylation analysis reveals local changes in resistant and susceptible soybean lines in response to *Phytophthora sansomeana*

**DOI:** 10.1093/g3journal/jkae191

**Published:** 2024-08-14

**Authors:** Charlotte N DiBiase, Xi Cheng, Gwonjin Lee, Richard C Moore, Austin G McCoy, Martin I Chilvers, Lianjun Sun, Dechun Wang, Feng Lin, Meixia Zhao

**Affiliations:** Department of Biology, Miami University, Oxford, OH 45056, USA; Plant Molecular and Cellular Biology Graduate Program, University of Florida, Gainesville, FL 32611, USA; Department of Microbiology and Cell Science, University of Florida, Gainesville, FL 32611, USA; Department of Microbiology and Cell Science, University of Florida, Gainesville, FL 32611, USA; Department of Biology, Miami University, Oxford, OH 45056, USA; Department of Plant, Soil and Microbial Sciences, Michigan State University, East Lansing, MI 48824, USA; Department of Plant, Soil and Microbial Sciences, Michigan State University, East Lansing, MI 48824, USA; College of Agronomy and Biotechnology, China Agricultural University, Beijing 100193, China; Department of Plant, Soil and Microbial Sciences, Michigan State University, East Lansing, MI 48824, USA; Department of Plant, Soil and Microbial Sciences, Michigan State University, East Lansing, MI 48824, USA; Fisher Delta Research, Extension, and Education Center, Division of Plant Sciences and Technology, University of Missouri, Portageville, MO 63873, USA; Plant Molecular and Cellular Biology Graduate Program, University of Florida, Gainesville, FL 32611, USA; Department of Microbiology and Cell Science, University of Florida, Gainesville, FL 32611, USA

**Keywords:** DNA methylation, differentially methylated regions, *Phytophthora sansomeana*, soybean, epigenetic responses, Plant Genetics and Genomics

## Abstract

*Phytophthora sansomeana* is an emerging oomycete pathogen causing root rot in many agricultural species including soybean. However, as of now, only one potential resistance gene has been identified in soybean, and our understanding of how genetic and epigenetic regulation in soybean contributes to responses against this pathogen remains largely unknown. In this study, we performed whole genome bisulfite sequencing (WGBS) on two soybean lines, Colfax (resistant) and Williams 82 (susceptible), in response to *P. sansomeana* at two time points: 4 and 16 hours post-inoculation to compare their methylation changes. Our findings revealed that there were no significant changes in genome-wide CG, CHG (H = A, T, or C), and CHH methylation. However, we observed local methylation changes, specially an increase in CHH methylation around genes and transposable elements (TEs) after inoculation, which occurred earlier in the susceptible line and later in the resistant line. After inoculation, we identified differentially methylated regions (DMRs) in both Colfax and Williams 82, with a predominant presence in TEs. Notably, our data also indicated that more TEs exhibited changes in their methylomes in the susceptible line compared to the resistant line. Furthermore, we discovered 837 DMRs within or flanking 772 differentially expressed genes (DEGs) in Colfax and 166 DMRs within or flanking 138 DEGs in Williams 82. These DEGs had diverse functions, with Colfax primarily showing involvement in metabolic process, defense response, plant and pathogen interaction, anion and nucleotide binding, and catalytic activity, while Williams 82 exhibited a significant association with photosynthesis. These findings suggest distinct molecular responses to *P. sansomeana* infection in the resistant and susceptible soybean lines.

## Introduction

Plant hosts recognize pathogens through pathogen/microbe-associated molecular patterns (PAMP/MAMP). PAMP/MAMP-triggered immunity (PTI) is triggered by the extracellular detection of exogenous elicitors and the internal recognition of endogenous elicitors in the form of damage-associated molecular patterns. Effector proteins secreted by the pathogen typically circumvent or interfere with PTI (Thomma *et al*. 2011). Effector molecules are recognized by nucleotide-binding domain, leucine-rich-repeat-containing receptors (NB-LRR), which initiate the effector-triggered immunity (ETI) response (Jones and Dangl 2006; Wu *et al*. 2018; Ngou *et al*. 2022). PTI and ETI work together to confer immunity against specific pathogens (Naveed *et al*. 2020; Ngou *et al*. 2021; Tena 2021).

In the *Phytophthora* genus, the high genetic diversity in elicitor and effector genes makes the pathogen highly adaptable, thus increasing its pathogenicity (Qutob *et al*. 2009; Derevnina *et al*. 2016; Yang *et al*. 2018a, 2018b). Elicitins are oomycete-specific PAMPs that act as the primary triggers for PTI upon infection (Derevnina *et al*. 2016). While PAMPs are typically conserved across species and individuals within a genus, elicitins exhibit remarkable diversity (Derevnina *et al*. 2016). *Phytophthora* effector genes also exhibit significant genetic variations, indicating the pathogen's ability to coevolve with potential hosts (Yang *et al*. 2018b). For example, in different strains of *P. sojae*, avirulence genes *Avr1a* and *Avr3a* show substantial copy number variation, leading to changes in virulence (Qutob *et al*. 2009).

Resistance to *P. sojae* (*Rps*) is effectively controlled by over 40 *Rps* genes/alleles in soybean (Lin *et al*. 2022). Many of these *Rps* genes belong to the NB-LRR family, which can recognize the pathogen effectors and trigger ETI (Jones and Dangl 2006; Wu *et al*. 2018; Ngou *et al*. 2022). *NB-LRR* genes often have high levels of inter- and intraspecific sequence and copy number variation due to unequal crossing-over within NB-LRR clusters (Kuang *et al*. 2004; McHale *et al*. 2006). Interestingly, many of these *NB-LRR* genes are targeted by microRNAs and generate secondary *trans*-acting small interfering RNAs (siRNAs) that regulate other genes potentially crucial for plant defense. However, the regulatory mechanism remains relatively poorly characterized (Zhai *et al*. 2011; Fei *et al*. 2013; Zhao *et al*. 2015).

In addition to genetic regulation, epigenetic regulation can influence both *Phytophthora* pathogenicity and host susceptibility (Pais *et al*. 2018; Shen *et al*. 2018; Wang *et al*. 2020). Epigenetic regulation encompasses various mechanisms, including DNA methylation, histone modification, and noncoding RNA (ncRNA). In plants, DNA methylation controls cellular processes by adding a methyl group to a cytosine base in one of three sequence contexts (CG, CHG, and CHH, where H represents A, T, or C). The initiation of de novo methylation at all three cytosine contexts is catalyzed by domains rearranged methyltransferase 2 (DRM2) through the canonical RNA-directed DNA methylation (RdDM) pathway (Law and Jacobsen 2010; Matzke and Mosher 2014). In the RdDM pathway, single-stranded RNA is transcribed by RNA polymerase IV (Pol IV) and then copied into double-stranded RNA by RNA-directed RNA polymerase 2 (RDR2). This double-stranded RNA is processed by Dicer-like 3 (DCL3) into 24-nucleotide (nt) siRNAs, which target the scaffold transcripts generated from RNA polymerase V (Pol V), triggering de novo methylation (Law and Jacobsen 2010; Matzke *et al*. 2015; Erdmann and Picard 2020). Once established, CG and CHG methylation can be maintained independently of siRNAs through DNA replication. However, the maintenance of methylation in the CHH context requires the continuous presence of siRNAs (Cuerda-Gil and Slotkin 2016; Liu and Zhao 2023; Liu *et al*. 2024).

Gene body methylation preferentially occurs in the CG context and is often associated with genes that are constitutively expressed (Takuno and Gaut 2012; Zhang *et al*. 2018; Muyle *et al*. 2022). Methylation in the promoter region of genes interferes with transcription factors and indirectly promotes repressive histone modifications, thereby inhibiting gene expression (Domcke *et al*. 2015; Zhu *et al*. 2016; Zhang *et al*. 2020). While methylation in the promoter region typically represses gene transcription, regions near transposon-gene boundaries exhibited high levels of CHH methylation and RdDM activity, which is found to be associated with transcriptionally active genes in maize (Gent *et al*. 2013; Li *et al*. 2015; Liu *et al*. 2024). Consequently, methylation in gene bodies, transposable elements (TEs), and their flanking regions can have diverse effects on gene expression. The contrasting influences across different genomic regions contribute to the complexity of methylation's effect on gene expression, which remains largely unresolved.

Pathogen-induced epigenetic changes, especially DNA methylation alterations, have been observed in a wide range of plant species (Dowen *et al*. 2012; Geng *et al*. 2019; Rambani *et al*. 2020; Wang *et al*. 2020; Huang and Jin 2021; Xiao *et al*. 2021). In *Arabidopsis*, plants with DNA methylation defects are found to be more resistant to the bacterial pathogen *Pseudomonas syringae* pv. *tomato* DC3000 (*Pst*) and demonstrate an elevated salicylic acid (SA)-dependent response (Dowen *et al*. 2012). In soybean, upon infection with soybean cyst nematodes, resistant lines exhibit enhanced global methylation levels in both genes and TEs, along with correlations between differentially methylated regions (DMRs) and known resistance loci in resistant individuals that are not observed in susceptible individuals (Rambani *et al*. 2020). Moreover, CHH methylation displays a more dynamic nature than CG and CHG methylation, with instances of hypomethylation observed post-inoculation (Geng *et al*. 2019; Xiao *et al*. 2021).

*P. sansomeana*, an oomycete pathogen, was distinguished from the *Phytophthora megasperma* complex as a causal agent of phytophthora root rot (PRR) across a broad spectrum of hosts, including soybean, corn, white clover, pea, carrots, and several others (Hansen *et al*. 2009; Zelaya-Molina *et al*. 2010; Rojas *et al*. 2017). Compared to *P. sojae*, *P. sansomeana* is significantly more virulent in reducing root growth in soybean seedlings (Alejandro Rojas *et al*. 2017). Despite its wide distribution, only two minor effect quantitative resistance loci and one potential resistance gene have been identified in soybean against *P. sansomeana* (Lin *et al*. 2021, 2024). Our previous research screened over 500 soybean germplasm and identified several resistant soybean lines to *P. sansomeana* (Lin *et al*. 2024). To understand the molecular responses and aid in the identification of resistance genes, we previously conducted comprehensive transcriptomic analyses at four time points (2, 4, 8, and 16 hpi) in two resistant (Colfax and NE2701) and two susceptible lines (Senaki and Williams 82). Our findings reveal minimal differentially expressed genes (DEGs) at 2 hpi across all lines, with over 5,000 DEGs at 16 hpi in Colfax (Lee *et al*. 2024). The DEGs in resistant lines are primarily associated with defense response, ethylene signaling, and reactive oxygen species-mediated defenses. Additionally, numerous differentially expressed TEs, mostly upregulated post-inoculation, were observed. Given that TE sequences are frequently silenced by epigenetic pathways that involve siRNAs, DNA methylation, and histone modification (Slotkin and Martienssen 2007; Liu and Zhao 2023), we sought to determine whether changes in TE expression are correlated with alterations in methylation levels and their potential impact on gene expression, which is crucial for understanding resistance mechanisms. Therefore, in this study, we performed whole genome bisulfite sequencing (WGBS) on Colfax, a stable resistant line in both greenhouse and field conditions, and Williams 82, a susceptible line chosen as the soybean reference genome. We focused on two critical time points (4 and 16 h post-pathogen and mock inoculation) identified from our prior RNA-seq findings, capturing early and late infection stages by *P. sansomeana* (Lee *et al*. 2024). Our data revealed that while no significant changes occurred in global DNA methylation levels after inoculation in both the resistant and susceptible lines, local methylation changes were observed. Notably, increased CHH methylation after inoculation occurred on and near genes and TEs at the early time point (4 hpi) in the susceptible line, while in the resistant line, this increase was observed later (16 hpi). Furthermore, more TEs exhibited changes in their methylomes in the susceptible line compared to the resistant line. Additionally, we identified DMRs that may affect the expression of flanking genes, potentially playing a role in soybean responses to *P. sansomeana*.

## Methods

### Selection of soybean lines and inoculation procedure

Two soybean lines, Colfax and Williams 82, were identified as resistant and susceptible, respectively, to the pathogen *P. sansomeana*. Colfax was identified as resistant to the pathogen through previous screening of over 500 soybean lines (Lin *et al*. 2024). Williams 82, being susceptible to the pathogen and serving as the reference genome for soybean, made it an ideal candidate for our analysis. Further confirmation of their respective phenotypes was obtained through subsequent field and greenhouse experiments involving control (mock inoculation) and treatment (inoculation with *P. sansomeana*) individuals.

Individual plants from each line were grown in the greenhouse at Michigan State University. *P. sansomeana* was cultured on lima bean agar following the method previously described (Dorrance *et al*. 2008; Lin *et al*. 2021; Lin *et al*. 2024). Ten seedlings of each line were challenged with *P. sansomeana* isolate *MPS17-22* using the standard hypocotyl inoculation method (Dorrance *et al*. 2008; Lin *et al*. 2021; Lin *et al*. 2024). Two time points were selected for tissue collection: 4 and 16 hours post-inoculation (hpi). At each time point, we performed four biological replicates for both pathogen-inoculated and mock-inoculated samples. In each replicate, we collected stem tissues from 7 to 8 seedlings by excising 2–3 cm across the wounded site, stored the samples immediately in liquid nitrogen, and subsequently preserved them at −80°C. The remaining seedlings were retained for evaluating symptom development, which was assessed 7 days post-inoculation.

DNA was extracted from the stem tissue of the preserved individuals for two of the four biological replicates (Supplementary Table 1). DNA extraction was carried out using the modified cetyltrimethylammonium bromide (CTAB) method (Guo *et al*. 2022). Nanodrop spectrometry in conjunction with gel electrophoresis was used to ensure the quality and quantity of the extracted DNA. These DNA were sent to Novogene (Novogene Corporation Inc., USA) for library construction and WGBS sequencing, where high-throughput paired-end reads with a length of 150 bp were generated.

### Read mapping and methylation analysis

Raw reads were quality controlled by FastQC. Paired-end reads were aligned to the Williams 82 v4 reference genome using Bismark, which uses bowtie2 for mapping, under the following parameters (-I 50, -N 1) (Krueger and Andrews 2011; Valliyodan *et al*. 2019). Deduplication was performed on the WGBS sequence data using the deduplication package under Bismark to remove PCR duplicates. The bismark2bedgraph and coverage2cytosine scripts in Bismark were used to extract methylated cytosines and count methylated and unmethylated reads following our previous research (Zhao *et al*. 2017, 2021; Yin *et al*. 2022). The relative methylation level at each cytosine was calculated using the following formula: total methylated reads/(total methylated reads + total unmethylated reads) covering that cytosine.

### Chromosome-wide methylation and methylation on and flanking protein-coding genes and TEs

To elucidate chromosome-wide methylation patterns, we divided each soybean chromosome into 500 kb windows with a 100 kb shift. Within each of these 500 kb windows, we calculated the average methylation level and plotted it across the entire chromosome. Methylation distribution across all known genes and TEs, as well as flanking regions 2 kb upstream and downstream of these genes and TEs, was characterized by separating each gene/TE into 40 equally sized bins and averaging methylation level across each bin-sized region. It is important to note that bin sizes vary along gene and TE bodies due to their variable lengths. These methylation distributions were combined to generate an average distribution of methylation across all known genes and TEs in the soybean genome between different lines, treatments, contexts, and time points (Schultz *et al*. 2012).

### Analysis of DMRs

The methylation proportion of each cytosine generated by Bismark was used to identify DMRs using metilene (v.0.23) (Jühling *et al*. 2016). We removed cytosines with no read coverage from our analysis. DMRs between treatment (pathogen-inoculated) and control (mock-inoculated) individuals were identified at both time points. DMRs were defined as genomic regions that were >300 bp apart with significantly different methylation levels between pathogen-inoculated and mock-inoculated individuals. Specially, a DMR was determined as containing a minimum of eight cytosine sites, with the distance of two adjacent cytosine sites <300 bp, and with the average methylation differences in CG and CHG >0.4 and in CHH >0.2 between treatment and control (Shen *et al*. 2018; Xu *et al*. 2020; Liu *et al*. 2024).

DMRs were compared in number and location between lines, time points, and treatments using a combination of bedtools and custom Python scripts. To gain insights into the genomic contexts of DMRs, the locations of DMRs were intersected with protein-coding genes and TEs, as well as 2 kb upstream and downstream regions of each gene and TE. The locations of DMRs were also intersected between different cytosine contexts within the same sample to understand how distinct methylation contexts overlap with each other within the same treatment group.

### Correlation of DMRs with DEGs

To identify DMRs potentially influencing gene expression, we focused on the DEGs containing DMRs within their gene bodies and within 2 kb flanking regions. To do so, we used the intersect function in bedtools between DMRs and the gene bodies and 2 kb upstream and downstream regions of the DEGs. The list of DEGs was obtained from our previous RNA sequencing (RNA-seq) experiment (Lee *et al*. 2024). To determine the biological categories of DEGs, GO enrichment analysis was conducted using g:GOst functional profiling in g:Profiler (Raudvere *et al*. 2019). Relative expression of genes involved in the RdDM pathway was calculated using log_2_(fold change) between lines and time points in inoculated individuals. The protein sequences of 79 genes in the RdDM pathway were collected from *Arabidopsis*, and BLASTP was used to find homologous genes in soybean.

## Results

### No significant changes in global DNA methylation levels after inoculation in both the resistant and susceptible lines

To understand the epigenetic responses of soybean to *P. sansomeana*, we performed WGBS on two soybean lines, Colfax and Williams 82, both before (control) and after (treatment) *P. sansomeana* inoculation, at two distinct time points (4 and 16 hpi) (Supplementary Table 1). Before inoculation, the overall methylation levels in all three cytosine contexts were slightly lower in Colfax compared to Williams 82 in the control samples, with CG 55.2% in Colfax versus 57.2% in Williams 82, CHG 35.9% versus 37.2%, and CHH 5.7% in the former versus 6.2% in the latter (Supplementary Fig. 1), indicating variations in the methylation levels in different soybean genetic backgrounds. Additionally, we plotted the methylation levels across the 20 soybean chromosomes. The chromosome-wide methylation patterns were consistent with the genome-wide data, with no significant differences in CG and CHG between the two lines (Supplementary Figs. 2–5). In contrast, the susceptible line, Williams 82, exhibited slightly higher levels of CHH methylation compared to the resistant line, Colfax (Supplementary Figs. 6 and 7). Next, we compared the methylation changes post-pathogen inoculation. In both the resistant and susceptible lines, no significant changes in global DNA methylation (chromosome-wide methylation) levels after inoculation were observed (Supplementary Fig. 1), suggesting that pathogen inoculation does not induce significant changes in the global methylation profiles of these two soybean lines.

### CHH methylation on and near protein-coding genes increases after inoculation in both lines but occurs earlier in the susceptible line

**Fig. 1.**
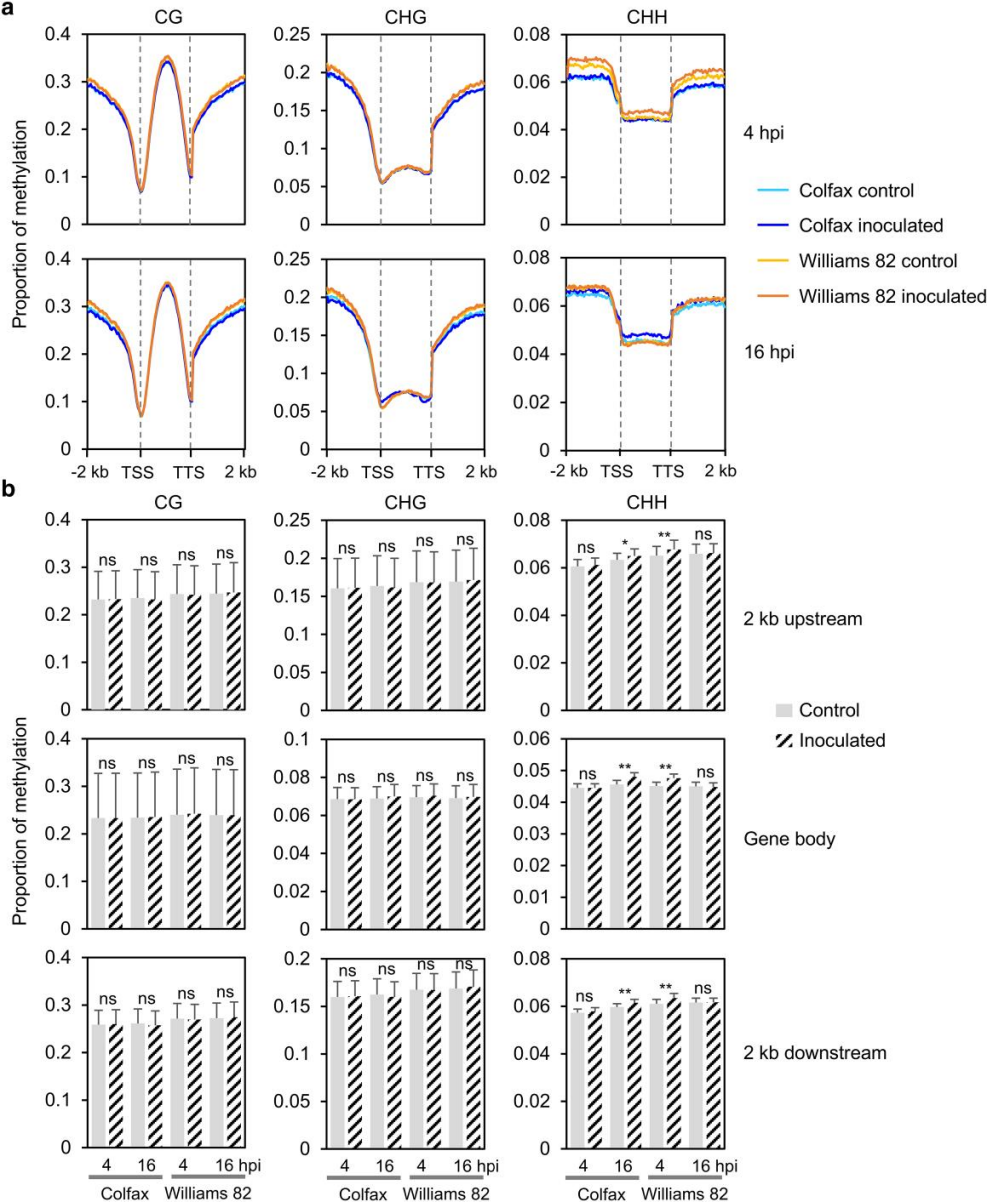
CHH methylation on and near genes significantly increases after inoculation in both lines but occurs earlier in the susceptible line. a) Patterns of methylation in and flanking protein-coding genes. b) Statistical analysis of methylation changes between control and inoculated samples. The statistical analysis was conducted by Student's *t* test. **P < 0.001; *P < 0.05; ns, not significant. DNA methylation levels were calculated in 50 bp windows in the 2 kb upstream and downstream regions of the genes. Each gene was divided into 40 equally sized bins to measure the gene body methylation. Bin sizes differ from gene to gene because of the different lengths of genes. Methylation for each sample was calculated as the proportion of methylated C over total C in each sequence context averaged for each window.

We next investigated the methylation changes on and near protein-coding genes. We plotted DNA methylation levels of CG, CHG, and CHH within gene bodies, 2 kb upstream of transcription start sites (TSSs), and 2 kb downstream of transcription termination sites (TTSs). Overall, at both 4 and 16 hpi, gene bodies exhibited higher CG but lower CHG and CHH methylation, except at the TSSs and TTSs, where methylation was typically very low (Fig. 1a). CG and CHG methylation within gene bodies did not show significant changes after inoculation in both lines (Fig. 1b). The levels of CG and CHG methylation in the 2 kb flanking regions of genes were lower in Colfax, aligning with the genome-wide methylation pattern, and remained relatively stable after inoculation in both the resistant and susceptible lines (Fig. 1b).

In the CHH context, at 4 hpi, Williams 82 was more methylated over gene bodies and their flanking regions relative to Colfax. Interestingly, at this time point, the methylation levels in the gene bodies and 2 kb flanking regions of genes in Williams 82 significantly increased after inoculation, while no obvious difference in CHH methylation was observed in Colfax after inoculation (Fig. 1a and b). By 16 hpi, CHH methylation levels in gene bodies and flanking regions had returned to a similar level between the control and inoculated samples of Williams 82. Conversely, at this time point, methylation levels significantly increased in Colfax after inoculation, particularly in the gene body regions (Fig. 1a and b). Overall, our data indicated that the susceptible line (Williams 82) exhibited increased CHH methylation at an earlier time point (4 hpi), while a similar pattern emerged later in the resistant line (Colfax), suggesting dynamic and differential epigenetic responses to the pathogen between these two lines.

### CHH methylation on and near TEs increases after inoculation in both lines

**Fig. 2.**
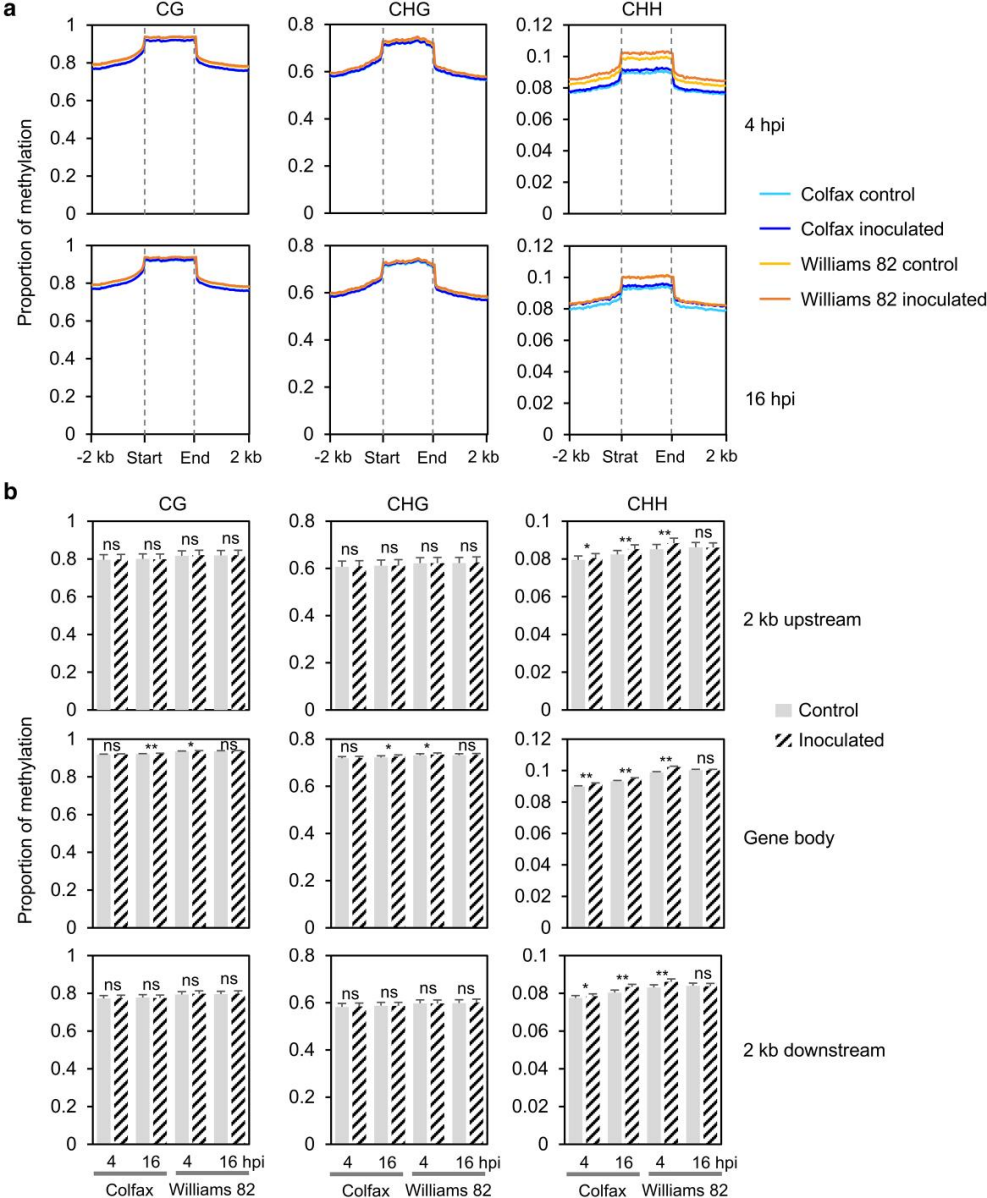
CHH methylation on and near transposable elements (TEs) significantly increases after inoculation in both lines. a) Patterns of methylation in and flanking TEs. b) Statistical analysis of methylation changes between control and inoculated samples. The statistical analysis was conducted by Student's *t* test. **P < 0.001; *P < 0.05; ns, not significant. DNA methylation levels were calculated in 50 bp windows in the 2 kb upstream and downstream regions of the TEs. Each TE was divided into 40 equally sized bins to measure the TE body methylation. Bin sizes differ from TE to TE because of the different lengths of TEs. Methylation for each sample was calculated as the proportion of methylated C over total C in each sequence context averaged for each window.

Given that TE sequences are frequently targeted by DNA methylation (Slotkin and Martienssen 2007; Liu and Zhao 2023), we examined the methylation changes on and near TEs following inoculation. Overall, at both 4 and 16 hpi, TEs exhibited high methylation levels throughout their bodies in all three cytosine contexts. For both CG and CHG, the methylation within TE bodies was nearly 30% higher than in flanking regions, and TEs displayed higher methylation levels compared to genes (Fig. 2a). Notably, no significant changes in CG and CHG methylation were observed following inoculation in the 2 kb flanking regions of TEs in either the resistant or susceptible lines (Fig. 2b). Interestingly, CG and CHG methylation within TE bodies significantly increased after inoculation in both lines, with earlier changes observed in the susceptible line, mirroring the pattern of CHH methylation in genes (Figs. 1b and 2b).

In the CHH context, Williams 82 consistently exhibited higher CHH methylation within TE bodies and their flanking regions than Colfax, aligning with the CHH methylation patterns observed for genes and genome-wide methylation (Figs. 1a and 2a). At 4 hpi, CHH methylation significantly increased across TEs and their flanking regions in both Colfax and Williams 82, although the degree of change was smaller in Colfax compared to Williams 82 at this time point (Fig. 2b). By 16 hpi, CHH methylation in Williams 82 had returned to levels similar to those observed in both control and inoculated samples. Interestingly, at this time point, a significant increase in CHH methylation was observed in both TE bodies and their 2 kb flanking regions post-inoculation in Colfax. Overall, our data demonstrated that CHH methylation increased in response to *P. sansomeana* in both genes and TEs, with this change occurring earlier in the susceptible line and later in the resistant line.

### The resistant and susceptible lines exhibit large local methylation differences, particularly in CHH methylation, in response to *P. sansomeana* infection

**Fig. 3.**
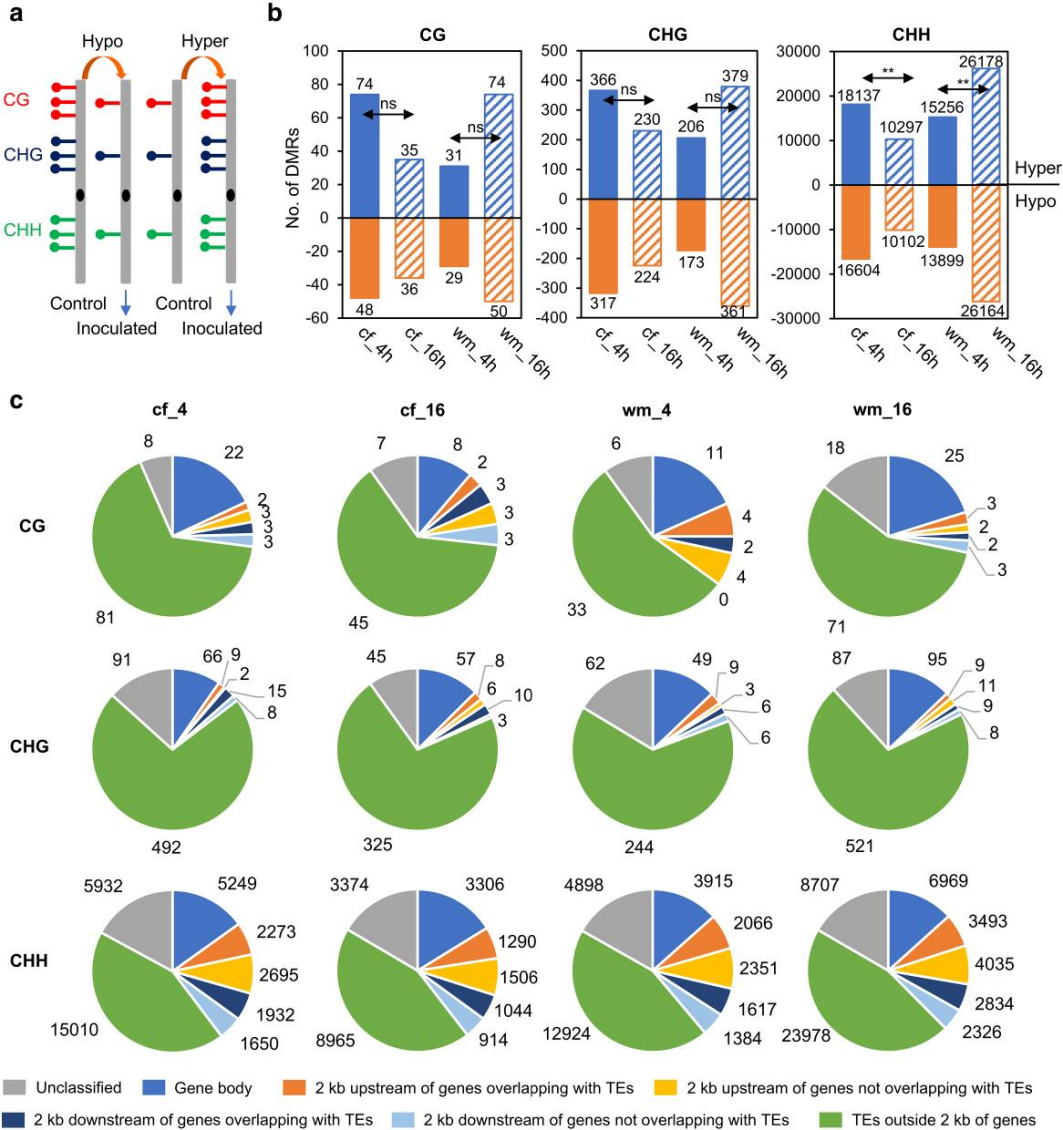
Resistant and susceptible lines exhibit large local methylation differences in response to *P. sansomeana* infection. a) Definition of hypo-DMRs (lower methylation after inoculation) and hyper-DMRs (higher methylation after inoculation) between mock (control) and pathogen-inoculated (treatment) samples. Red, blue, and green dots represent CG, CHG, and CHH methylation, respectively. b) DMR numbers decrease in Colfax but increase in Williams 82 following the time points. The statistical analysis was conducted by the *χ*^2^ test. **P < 0.001; ns, not significant. c) Genomic distribution of CG, CHG, and CHH DMRs. The positions of the DMRs were compared to the positions of genes and transposable elements (TEs) to determine their genomic distribution. The category “2 kb upstream and downstream of genes overlapping with TEs” indicates that the DMRs overlap with TEs within the 2 kb flanking regions of genes.

To identify genomic regions with local methylation changes, we identified differentially methylated regions (DMRs) following inoculation. These DMRs were categorized as hypermethylated or hypomethylated DMRs, indicating increased or decreased methylation levels after inoculation (Fig. 3a). In Colfax at 4 hpi, we identified 122 CG, 683 CHG, and 34,741 CHH DMRs, with 52.2–60.7% of them being hypermethylated (Fig. 3b). CHH DMRs, with the average length of 93 bp, were generally shorter than CG and CHG DMRs, which averaged 227 and 315 bp, respectively (Supplementary Fig. 8). In Colfax at 16 hpi, the numbers of DMRs (71 CG, 454 CHG, and 20,399 CHH) at all three cytosine contexts decreased for both hyper- and hypo-DMRs compared to the numbers of DMRs identified at 4 hpi. Interestingly, we observed the opposite trend with respect to the numbers of DMRs in Williams 82, where we detected a total of 53,206 DMRs at 16 hpi, significantly more than the 29,594 DMRs identified at 4 hpi (Fig. 3b). These CG, CHG, and CHH DMRs were largely distributed independently throughout the genome, with only a few of them overlapping with each other (Supplementary Fig. 9).

Next, we examined the locations of these DMRs relative to genes and TEs. At 4 hpi in Colfax, out of the 122 CG DMRs, 33 (27.0%) were located in genes or within the 2 kb flanking regions of genes, while 81 (66.4%) were within TEs outside the 2 kb flanking regions of genes (Fig. 3c). For CHG DMRs at the same time point in Colfax, a smaller proportion (14.6%, 100 out of 683) were found in genic and flanking regions, while the majority were in TEs outside 2 kb flanking regions of genes (72.0%, 492 out of 683). In contrast, CHH DMRs (39.7%, 13,799 out of 34,741) were predominantly enriched within and near genes, particularly within the 2 kb flanking regions of genes (Fig. 3c). The proportion of CHH DMRs (43.2%) within TEs outside 2 kb flanking regions of genes was lower than that of CG and CHG DMRs in the same category. At 16 hpi in Colfax and both time points in Williams 82, the percentages of the genomic locations of these DMRs, including both hyper- and hypo-DMRs, were very similar to 4 hpi (Fig. 3c, Supplementary Figs. 10 and 11). These findings highlight the substantial differences in local methylation induced by *P. sansomeana* infection between these two lines.

### More transposons exhibit changes in their methylomes in the susceptible line compared to the resistant line

**Fig. 4.**
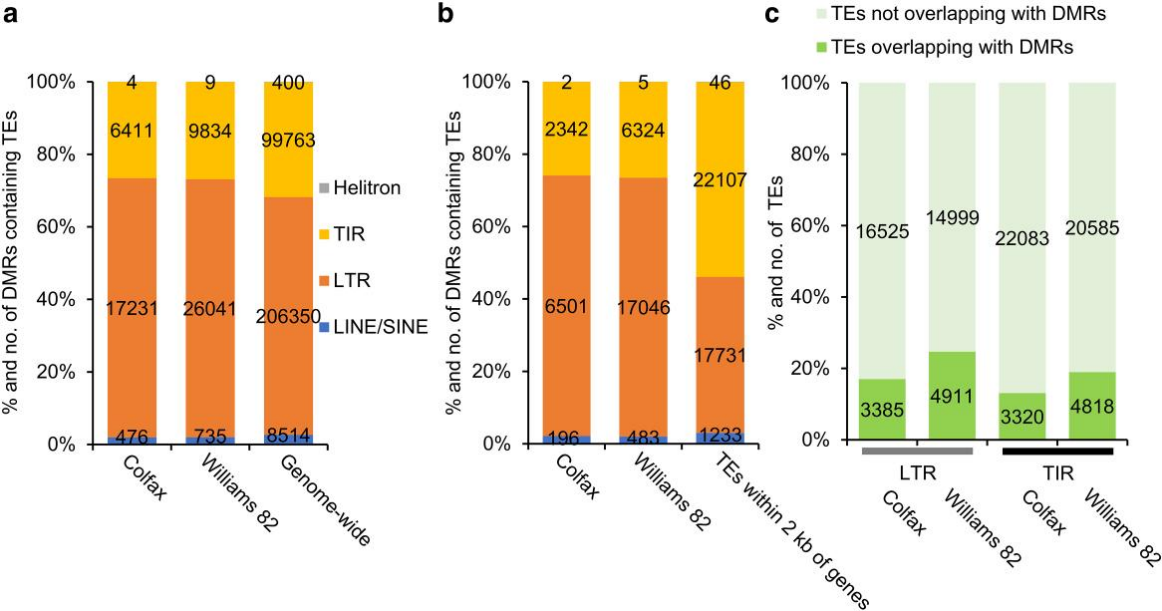
More transposons change their methylomes in the susceptible line compared to the resistant line. a) DMRs overlap with TEs outside the 2 kb flanking regions of genes. Genome-wide TEs that do not overlap with DMRs are used as a control here. b) DMRs overlap with TEs within the 2 kb flanking regions of genes. TEs within the 2 kb flanking regions of genes that do not overlap with DMRs are used as a control here. c) TEs overlap with DMRs within the 2 kb flanking regions of genes between Colfax and Williams 82. TIR, terminal inverted repeat DNA transposons; LTR, long terminal repeat retrotransposons; LINE, long interspersed nuclear element; SINE, short interspersed nuclear element.

Given that a substantial proportion (55.3–75.6%) of DMRs overlap with TEs, we wanted to identify which types of TEs show significant methylation changes following inoculation. We first examined the DMRs that overlapped with TEs located outside the 2 kb flanking regions of genes. Our data revealed that among these TEs, long terminal repeat (LTR) retrotransposons were the most abundant ones that exhibited the most notable changes in their methylomes in both Colfax and Williams 82, followed by terminal inverted repeat (TIR) DNA transposons. Interestingly, the proportions of DMRs overlapping with LTR retrotransposons in both lines were higher than the genome-wide proportion of LTR retrotransposons (Fig. 4a).

When focusing on DMRs overlapping with TEs within the 2 kb flanking regions of genes, we found that DMRs overlapping with LTR elements, particularly LTR-*Copia* retrotransposons, were significantly enriched in both Colfax (71.9%) and Williams 82 (71.9%), compared to the overall proportion of LTR elements within the 2 kb flanking regions of genes (43.1%) (Fig. 4b). This indicates that LTR elements within the 2 kb flanking regions of genes undergo substantial methylation changes in response to *P. sansomeana* infection. It is worth noting that many TEs contain multiple DMRs, so we next focused on TE elements that change methylome after inoculation. Out of the 19,910 LTR retrotransposons within the 2 kb flanking regions of genes, 4,911 (24.7%) overlapped with DMRs in Williams 82, significantly more than in Colfax (17.0%) (Fig. 4c). Further analysis of the time points revealed that these TEs predominantly altered their methylomes at 16 hpi, with fewer changes at 4 hpi (Supplementary Fig. 1^2^). A similar pattern was observed for TIR transposons, with Williams 82 showing a higher proportion of TIR elements (20.0%) overlapping with DMRs compared to Colfax (13.1%) (Fig. 4c and Supplementary Fig. 1^2^). Together, our data indicate that more transposons undergo methylation changes in Williams 82 compared to Colfax, suggesting that the resistant line exhibits greater stability than the susceptible line in response to the pathogen.

### Distinct molecular responses in the resistant and susceptible lines

**Fig. 5.**
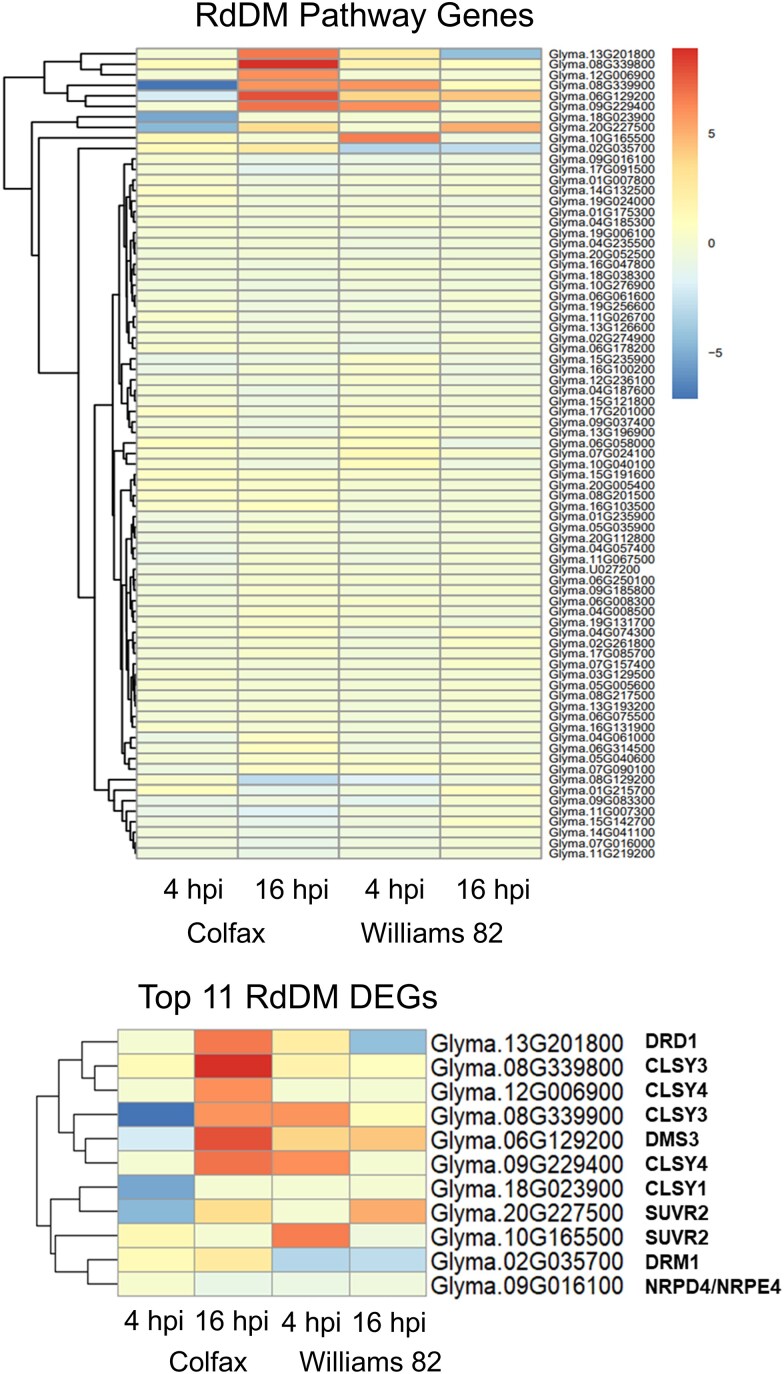
CLASSY family genes are upregulated following inoculation. Log_2_(fold change) of normalized expression values of 79 genes involved in the RdDM pathway was calculated between mock and pathogen-inoculated samples.

As de novo methylation is triggered by the RdDM pathway (Matzke and Mosher 2014; Matzke *et al*. 2015; Erdmann and Picard 2020), we wanted to determine whether the increased CHH methylation following inoculation was attributable to the increased expression of genes involved in this pathway. We initially compiled a list of genes associated with the RdDM pathway in *Arabidopsis* (Matzke and Mosher 2014; Matzke *et al*. 2015; Erdmann and Picard 2020; Liu and Zhao 2023) and performed a search within the soybean genome, resulting in the identification of 79 homologous genes. Subsequently, we analyzed the expression patterns of these 79 genes in our RNA-seq data and found that 8 genes were upregulated and 6 genes were downregulated after inoculation (Fig. 5) (Lee *et al*. 2024). Interestingly, five of the eight upregulated genes belonged to the *CLASSY* (*CLSY*) gene family, including *CLSY 3* and *4* (Fig. 5). Recent research has shown that CLSY proteins play a role in controlling tissue-specific methylation patterns in *Arabidopsis* (Zhou *et al*. 2022). *CLSY 3* and *4* were upregulated at 16 hpi in Colfax, whereas in Williams 82, their upregulation occurred at 4 hpi in Williams 82, consistent with the earlier increase in CHH methylation observed in Williams 82 (Figs. 1 and 2). This suggests that the RdDM pathway is activated earlier in the susceptible line compared to the resistant lines.

**Fig. 6.**
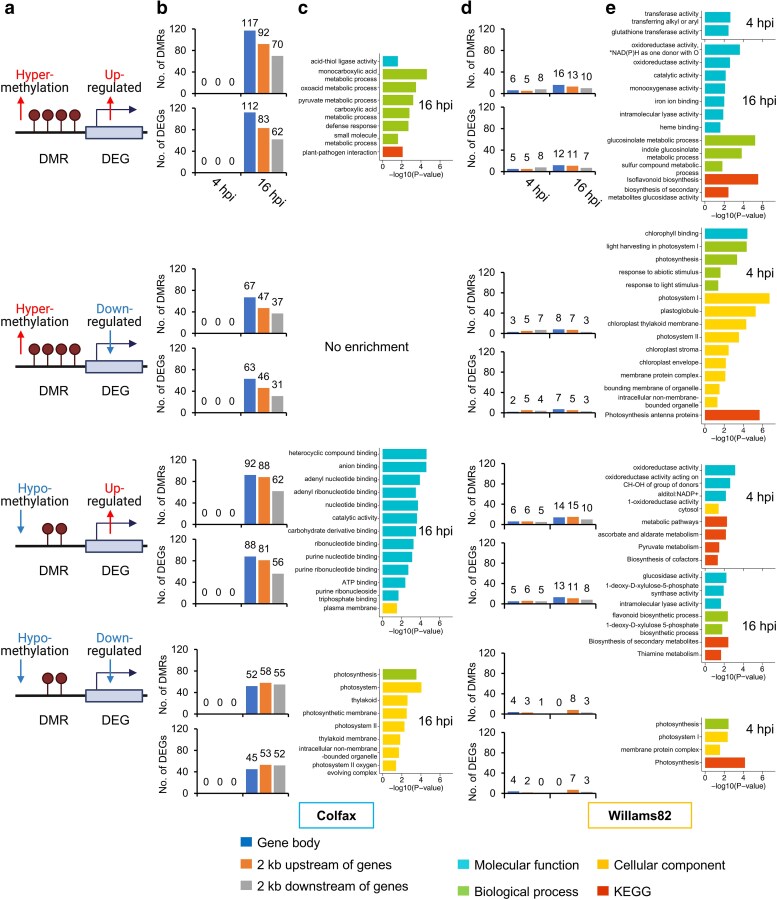
Association analysis of DMRs and their flanking DEGs. a) Four hypothetical models of the association between DMRs and DEGs. b) Numbers of DEGs that fit the hypothetical models in a) in Colfax. c) GO and KEGG enrichment analysis of the DEGs in Colfax at 16 hpi. d) Numbers of DEGs that fit the hypothetical models in a) in Williams 82. e) GO and KEGG enrichment analysis of the DEGs in Williams 82 at 4 and 16 hpi. GO enrichment analysis for c) and e) was conducted using g:GOSt functional profiling in g:Profiler (Raudvere *et al*. 2019). The figures were generated in *R*.

Next, we sought to determine whether these DMRs influenced the expression of flanking genes. We focused on the DMRs on or within the 2 kb flanking regions of DEGs. In Colfax, we identified 837 DMRs within or flanking 772 DEGs, a substantially higher number compared to Williams 82, which had 166 DMRs within or flanking 138 DEGs (Fig. 6 and Supplementary Table 2). It is worth noting that the majority (99.2%) of these DMRs were CHH DMRs. We next categorized these DMRs and DEGs into four categories: (1) hypermethylation of DMRs with upregulation of DEGs, (2) hypermethylation of DMRs with downregulation of DEGs, (3) hypomethylation of DMRs with upregulation of DEGs, and (4) hypomethylation of DMRs with downregulation of DEGs (Fig. 6a). In Colfax, DEGs overlapping with DMRs were only observed at 16 hpi across all four categories (Fig. 6b). In contrast, Williams 82 had fewer DEGs near DMRs, distributed at both 4 and 16 hpi (Fig. 6d). To gain further insights into the function of these DEGs, we conducted Gene Ontology (GO) and Kyoto Encyclopedia of Genes and Genomes (KEGG) analyses. In Colfax at 16 hpi, the upregulated DEGs were primarily associated with metabolic process, defense response, plant and pathogen interaction, anion and nucleotide binding, and catalytic activity (Fig. 6c). In contrast, in Williams 82 at 4 hpi, downregulated DEGs were mainly involved in photosynthesis-related functions (Fig. 6e), indicating distinct molecular responses to *P. sansomeana* infection between the resistant and susceptible lines.

## Discussion

### Lower levels of CHH methylation in the resistant line are likely associated with the disease response

In this study, we demonstrated the global and local methylation changes of two soybean lines with resistance and susceptibility to the oomycete pathogen *P. sansomeana*. Both before and after inoculation, we did not observe significant differences in methylation levels in CG and CHG contexts between the two lines, at both chromosome-wide and on genes or TEs (Figs. 1 and 2, and Supplementary Figs. 2–5). The lack of differential methylation visible chromosome-wide in the CG and CHG cytosines can likely be attributed to the global high CG and CHG methylation levels, which play crucial roles in preserving genome stability and regulating key genes (Kato *et al*. 2003; Lang *et al*. 2017; Liu *et al*. 2024). In addition, CG and CHG methylation are heritable and tend to be relatively stable across generations, making them less prone to dramatic changes in response to pathogen infection (Rambani *et al*. 2020).

However, CHH methylation was observed to be higher in the susceptible line even before inoculation, especially on and flanking genes and TEs (Figs. 1 and 2, Supplementary Figs. 6 and 7). This distinction is likely due to the different genetic backgrounds, rather than being directly related to the disease response. Interestingly, an increase in CHH methylation in the 2 kb flanking regions and bodies of both genes and TEs after inoculation was detected earlier in Williams 82 but later in Colfax (Figs. 1 and 2). This suggests that methylation changes are notably dynamic over time, with distinct variations observed between the resistant and susceptible lines. We do not attribute this solely to a background effect since our comparisons were made between the inoculated individuals and the control samples (mock inoculation) within the same genetic backgrounds. Instead, these findings imply that the epigenome of the resistant line remains more stable in response to pathogen infection, potentially contributing to its ability to resist the disease.

Hyper- or hypomethylation in response to biotic stresses has been observed in several plant species (Dowen *et al*. 2012; Yu *et al*. 2013; Lopez Sanchez *et al*. 2016; Geng *et al*. 2019; Tirnaz and Batley 2019; Rambani *et al*. 2020; Huang and Jin 2021). For instance, susceptible soybean lines exhibit reduced methylation levels, while resistant lines display increased methylation levels when challenged with cyst nematode (Rambani *et al*. 2020). It is important to note that cyst nematode and *P. sansomeana* are distinct pathogens, making a direct comparison of their epigenetic responses challenging. However, both pathogens induce methylation changes albeit in different patterns, underscoring the prevalence of epigenetic alterations in response to various biotic stresses. Moreover, plants with deficiencies in DNA methylation, such as *met1*, *drm1/drm2/cmt3* (*ddc*), *nrpd2* (the second subunit of Pol IV and Pol V), *nrpd1* (Pol IV), and *nrpe1* (Pol V), are more resistant to the bacterial pathogen *Pseudomonas syringae* pv. *tomato* DC3000 (*Pst*), which is associated with the enhanced SA-dependent response (Lopez *et al*. 2011; Dowen *et al*. 2012; Yu *et al*. 2013). The *nrpe1* mutants are also more resistant to the biotrophic oomycete pathogen *Hyaloperonospora arabidopsidis* (*Hpa*) but exhibit susceptibility to the necrotrophic pathogen *Plectosphaerella cucumerina*, which is associated with repressed sensitivity of jasmonic acid (JA)-inducible gene expression (Lopez Sanchez *et al*. 2016). It is possible that the increased methylation observed in the susceptible line represents a molecular strategy employed by the susceptible individuals in response to *P. sansomeana* infection. Considering that *P. sansomeana* is a recently identified pathogen, our understanding of the molecular and physiological mechanisms governing defense or stress responses to this pathogen remains largely incomplete.

### CHH methylation buffers the effects of pathogen stress on TE activation

Pathogen attacks may induce rapid genomic and epigenomic changes, including alteration of expression of TEs and genes, activation of endogenous retroviruses, and epigenetic reprogramming (Secco *et al*. 2015). We hypothesize that CHH methylation may buffer the global effects of pathogen attacks on transcriptional activation of TEs (the main targets of DNA methylation) in the genome, resulting in the increases in de novo CHH methylation after inoculation. Unlike CG and CHG cytosines, which are methylated at a high level, the level of CHH methylation is low, only 6% genome-wide (Supplementary Fig. 1). Despite the low level of CHH methylation, CHH cytosines are remarkably abundant in the soybean genome. Across the 20 soybean chromosomes, there are a total of 326,006,099 cytosines, out of which 9.4 and 11.8% are CG and CHG cytosines and 78.8% are CHH cytosines. Such high abundance makes CHH cytosines reasonable candidates for buffering the global impact of environmental stresses such as pathogen attacks on transcriptional activation of TEs to maintain genome stability. The dynamic changes of CHH methylation have been observed in many plants in response to both abiotic and biotic stresses (Geng *et al*. 2019; Guo *et al*. 2021; Xiao *et al*. 2021; Liu *et al*. 2024), suggesting that the buffer effects of CHH methylation are global and not specific for different stressors.

### DMRs largely do not overlap with DEGs despite differential expression of the RdDM genes

As CHH methylation exhibited the most significant differences between the two lines and is initiated by RdDM, we explored whether the genes involved in the RdDM pathway were differentially expressed (Matzke and Mosher 2014; Cuerda-Gil and Slotkin 2016; Erdmann and Picard 2020). While most RdDM genes were expressed similarly between the resistant and susceptible lines and at different time points (Fig. 5), the top 11 DEGs revealed intriguing patterns. In the resistant line, DEGs in the RdDM pathway were downregulated at 4 hpi and subsequently upregulated at 16 hpi, aligning with our hypothesis that CHH methylation can act as a buffer to alleviate stress induced by the pathogen. Among these top DEGs, five were members of the CLASSY gene family, known for its role in mediating tissue-specific methylation in *Arabidopsis* (Fig. 5) (Zhou *et al*. 2022). The differential expression of these genes further supports that methylation patterns related to disease resistance may be more specific at the levels of genes and tissues.

Surprisingly, there was a lack of substantial overlap between DMRs and DEGs, a phenomenon previously observed in response to various stressors in crops (Rambani *et al*. 2020; Tian *et al*. 2021). Despite an almost complete lack of correlation between DMRs and DEGs in the CG and CHG contexts, a small proportion (0.7%) of DMRs in the CHH context coincided with DEGs (Fig. 6). Interestingly, hypermethylated and hypomethylated CHH DMRs were associated with both upregulated and downregulated DEGs (Fig. 6). Hypomethylation in the promoter regions of genes can increase chromatin accessibility and recruitment of transcription factors and other proteins to the regions, leading to gene activation (Domcke *et al*. 2015; Zhu *et al*. 2016; Zhang *et al*. 2020). However, in maize, CHH methylation at “mCHH islands” has been found to be associated with transcriptionally active genes (Gent *et al*. 2013; Li *et al*. 2015; Liu *et al*. 2024). These islands have been proposed to serve as boundaries between highly deep heterochromatin and more active euchromatin to reinforce silencing of TEs located near genes (Gent *et al*. 2013; Li *et al*. 2015; Martin *et al*. 2021). In our recent research, CHH methylation can be associated with both enhanced or suppressed expression of flanking genes, in which we hypothesize that whether CHH methylation promotes or suppresses flanking gene expression is largely dependent on the histone modifications (e.g. H3K9me2 and H3K27me3) and histone variants (e.g. H2A.W) at these regions (To *et al*. 2020; Liu *et al*. 2024). It would be interesting to investigate the histone modifications or variants at these DMRs to further understand the coordination between DNA methylation and histone modifications in response to pathogen attacks.

It is worth noting that although we detected upregulated DEGs in Colfax that were primarily associated with metabolic processes, defense response, plant–pathogen interaction, anion and nucleotide binding, and catalytic activity (Fig. 6c), we do not believe these changes in gene expression are directly attributed to changes in methylation on or flanking these genes. In our RNA-seq analysis (Lee *et al*. 2024), we observed similar genes that do not have DMRs nearby, suggesting that the changes in methylation may be a consequence rather than a cause. Additionally, only two time points (4 and 16 hpi) were investigated in this study, as representatives of early and late responses to pathogen infection. However, given this is the first DNA methylation analysis of soybean in response to *P. sansomeana*, the real progress may be beyond this period. Future research encompassing additional time points may provide deeper insights into the genetic and epigenetic regulation mechanisms that govern disease resistance in soybean.

## Conclusion

In this study, we identified global and local methylation changes in soybean lines with resistance and susceptibility to *P. sansomeana* and determined their impact on the activation and suppression of nearby gene expression. Our data highlighted the significance of CHH methylation in the overall response to biotic stresses within both resistant and susceptible lines. The distinctions in CHH methylation levels between these lines underscore the contribution of the RdDM pathway to disease response, emphasizing the need for further investigation into the intricate interplay between methylation and gene expression in response to stress. Our findings provide valuable insights into the potential mechanisms underlying resistance to *P. sansomeana*.

## Supplementary Material

jkae191_Supplementary_Data

## Data Availability

The raw and processed data of mRNA and whole genome bisulfite sequencing presented in this study have been deposited in NCBI Gene Expression Omnibus under the accession number GSE240966 (Lee *et al*. 2024). Supplemental material available at G3 online.
