# Supplementary material for: DNA methylation analysis reveals local changes in resistant and susceptible soybean lines in response to Phytophthora sansomeana
Source: G3 (Bethesda). 2024 Aug 14;14(10):jkae191. doi: 10.1093/g3journal/jkae191 (PMC11457093; doi:10.1093/g3journal/jkae191)
Supplement: jkae191_Supplementary_Data [file jkae191_supplementary_data.zip › Supplementary_Figures_G3-2024-405239.pptx]

## Slide 1
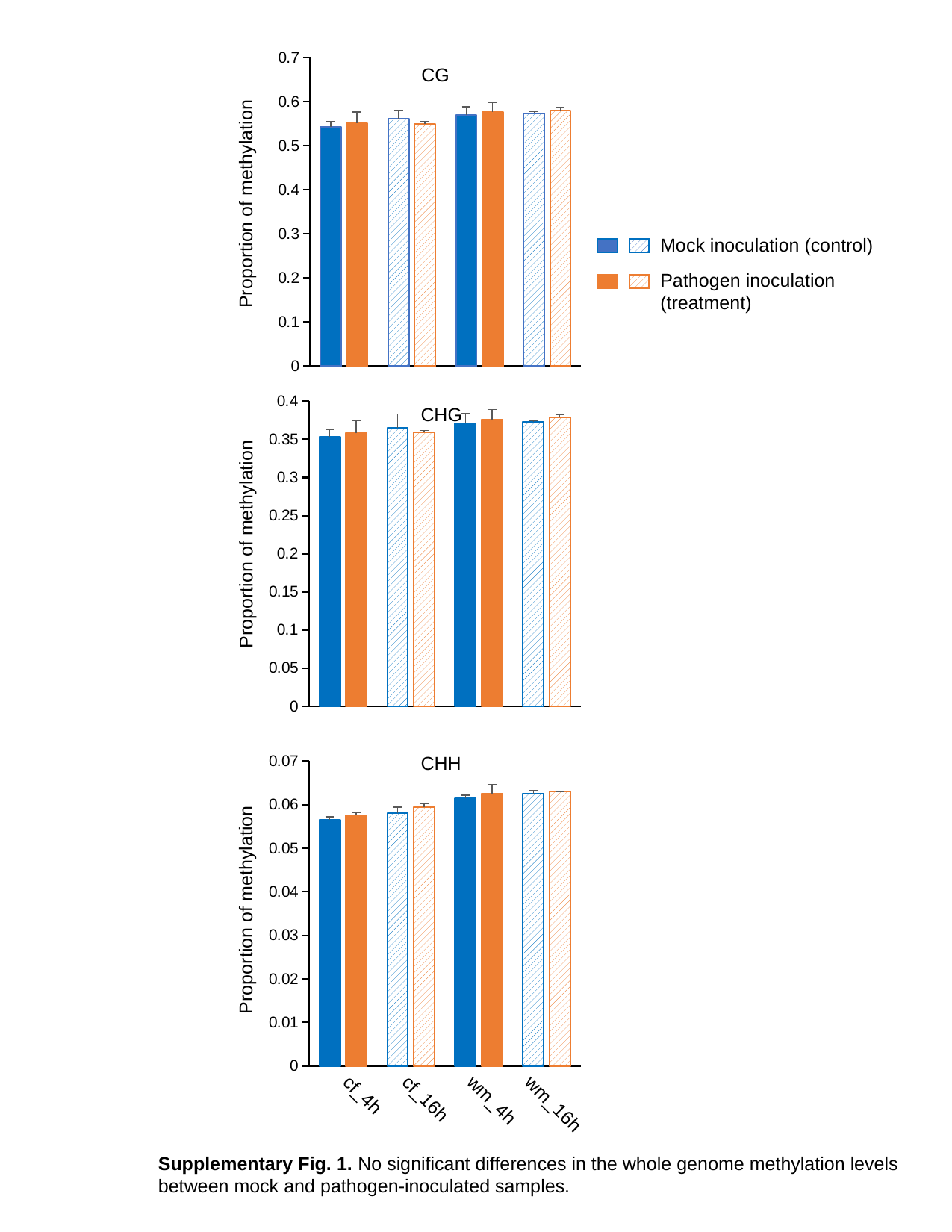

### Chart
| Category | | |
|---|---|---|CG
Proportion of methylation
Mock inoculation (control)
Pathogen inoculation (treatment)
### Chart
| Category | | |
|---|---|---|CHG
Proportion of methylation
### Chart
| Category | | |
|---|---|---|CHH
Proportion of methylation
cf_4h
cf_16h
wm_4h
wm_16h
Supplementary Fig. 1. No significant differences in the whole genome methylation levels between mock and pathogen-inoculated samples.

## Slide 2
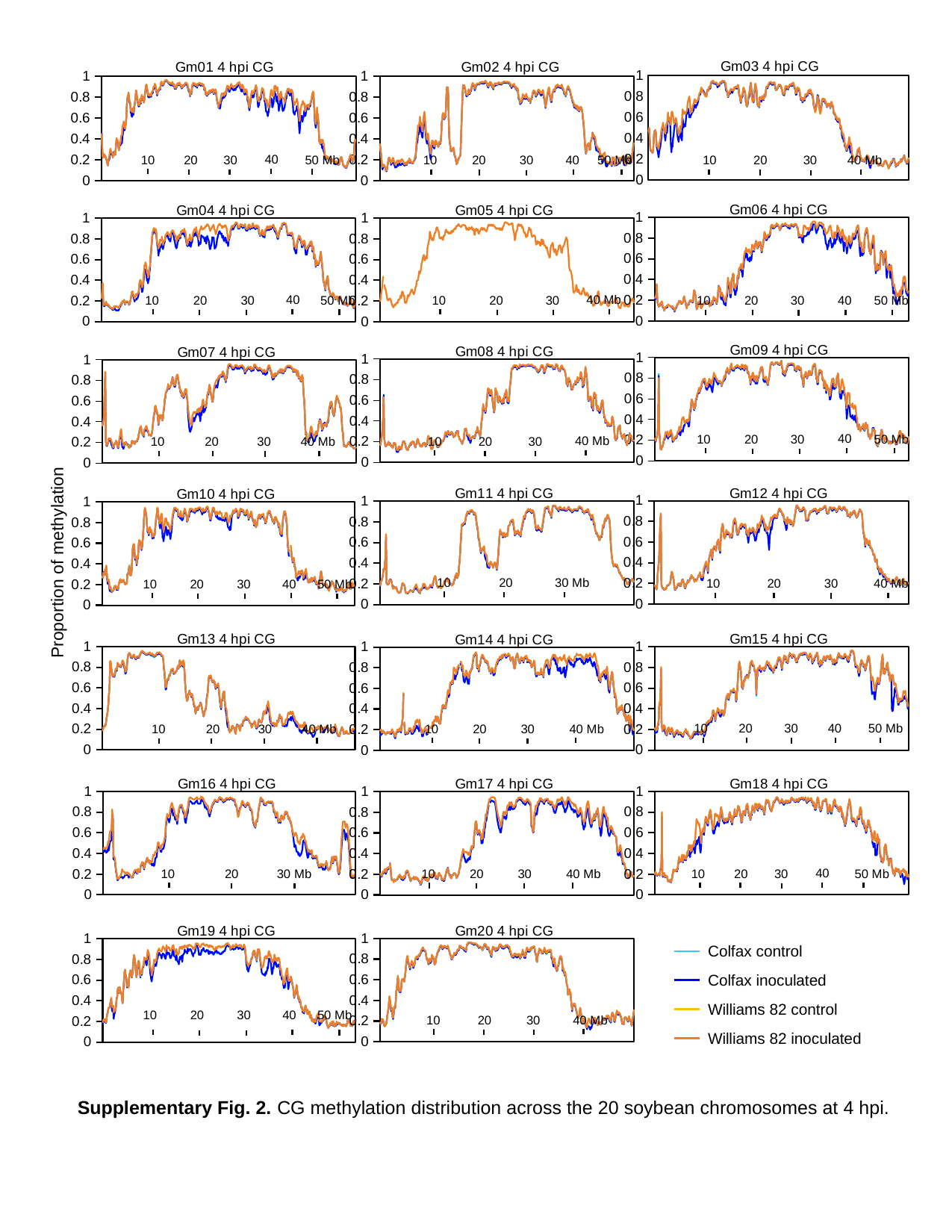

### Chart: Gm03 4 hpi CG
| Category | | | | |
|---|---|---|---|---|
### Chart: Gm01 4 hpi CG
| Category | cf_4_CG_c | cf_4_CG_t | wm_4_CG_c | wm_4_CG_t |
|---|---|---|---|---|
### Chart: Gm02 4 hpi CG
| Category | | | | |
|---|---|---|---|---|40
30
10
20
50 Mb
40
30
10
20
50 Mb
40 Mb
30
10
20
### Chart: Gm06 4 hpi CG
| Category | | | | |
|---|---|---|---|---|
### Chart: Gm04 4 hpi CG
| Category | | | | |
|---|---|---|---|---|
### Chart: Gm05 4 hpi CG
| Category | | | | |
|---|---|---|---|---|40
30
10
20
50 Mb
40 Mb
30
10
20
40
30
10
20
50 Mb
### Chart: Gm09 4 hpi CG
| Category | | | | |
|---|---|---|---|---|
### Chart: Gm08 4 hpi CG
| Category | | | | |
|---|---|---|---|---|
### Chart: Gm07 4 hpi CG
| Category | | | | |
|---|---|---|---|---|40
30
10
20
50 Mb
40 Mb
30
10
20
40 Mb
30
10
20
### Chart: Gm12 4 hpi CG
| Category | | | | |
|---|---|---|---|---|
### Chart: Gm11 4 hpi CG
| Category | | | | |
|---|---|---|---|---|
### Chart: Gm10 4 hpi CG
| Category | | | | |
|---|---|---|---|---|Proportion of methylation
30 Mb
10
20
40 Mb
30
10
20
40
30
10
20
50 Mb
### Chart: Gm13 4 hpi CG
| Category | | | | |
|---|---|---|---|---|
### Chart: Gm15 4 hpi CG
| Category | | | | |
|---|---|---|---|---|
### Chart: Gm14 4 hpi CG
| Category | | | | |
|---|---|---|---|---|40
30
10
20
50 Mb
40 Mb
30
10
20
40 Mb
30
10
20
### Chart: Gm16 4 hpi CG
| Category | | | | |
|---|---|---|---|---|
### Chart: Gm18 4 hpi CG
| Category | | | | |
|---|---|---|---|---|
### Chart: Gm17 4 hpi CG
| Category | | | | |
|---|---|---|---|---|40
30
10
20
50 Mb
40 Mb
30
10
20
30 Mb
10
20
### Chart: Gm20 4 hpi CG
| Category | | | | |
|---|---|---|---|---|
### Chart: Gm19 4 hpi CG
| Category | | | | |
|---|---|---|---|---|Colfax control
Colfax inoculated
Williams 82 control
Williams 82 inoculated
40
30
10
20
50 Mb
40 Mb
30
10
20
Supplementary Fig. 2. CG methylation distribution across the 20 soybean chromosomes at 4 hpi.

## Slide 3
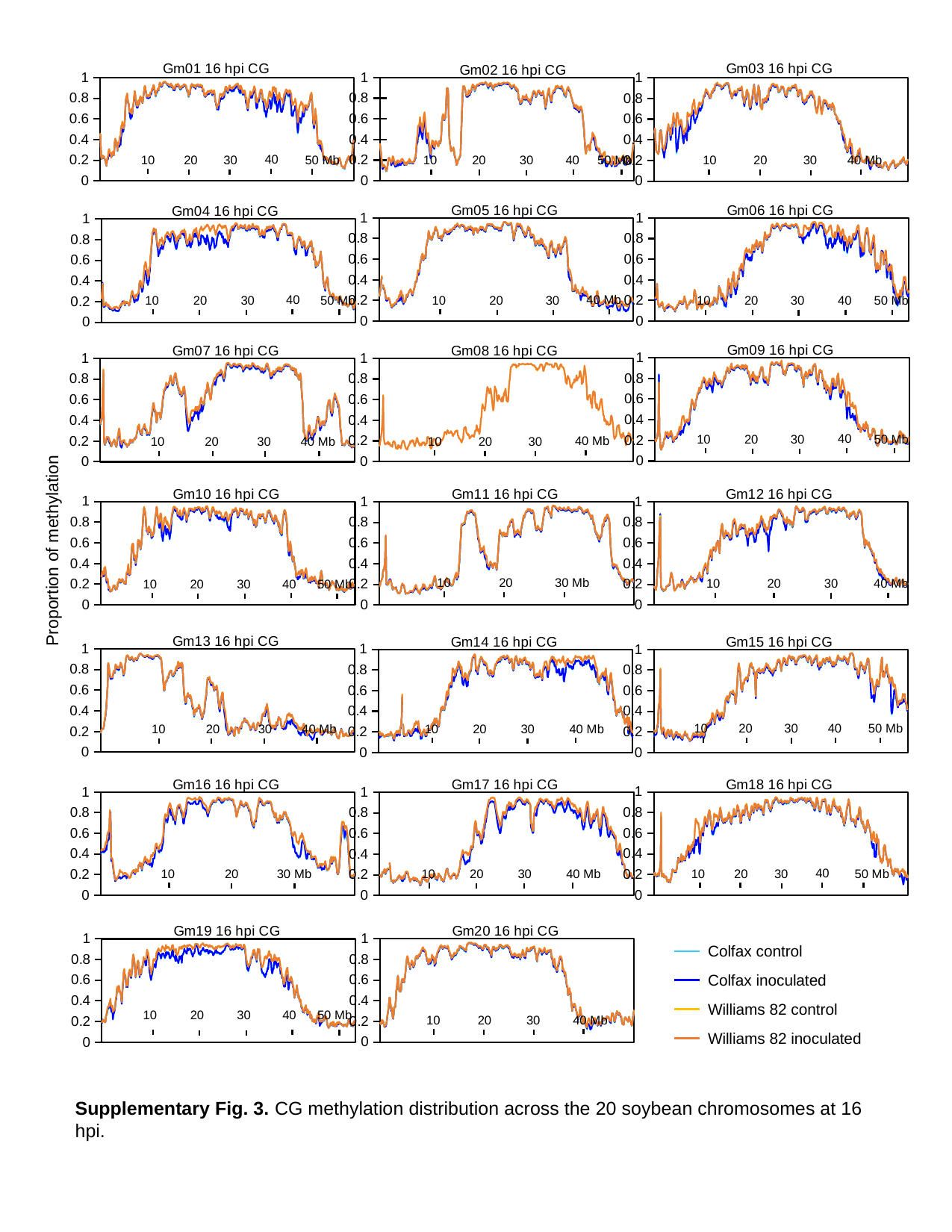

### Chart: Gm02 16 hpi CG
| Category | | | | |
|---|---|---|---|---|
### Chart: Gm01 16 hpi CG
| Category | cf_16_CG_c | cf_16_CG_t | wm_16_CG_c | wm_16_CG_t |
|---|---|---|---|---|
### Chart: Gm03 16 hpi CG
| Category | | | | |
|---|---|---|---|---|40
30
10
20
50 Mb
40
30
10
20
50 Mb
40 Mb
30
10
20
### Chart: Gm05 16 hpi CG
| Category | | | | |
|---|---|---|---|---|
### Chart: Gm06 16 hpi CG
| Category | | | | |
|---|---|---|---|---|
### Chart: Gm04 16 hpi CG
| Category | | | | |
|---|---|---|---|---|40
30
10
20
50 Mb
40 Mb
30
10
20
40
30
10
20
50 Mb
### Chart: Gm09 16 hpi CG
| Category | | | | |
|---|---|---|---|---|
### Chart: Gm08 16 hpi CG
| Category | | | | |
|---|---|---|---|---|
### Chart: Gm07 16 hpi CG
| Category | | | | |
|---|---|---|---|---|40
30
10
20
50 Mb
40 Mb
30
10
20
40 Mb
30
10
20
### Chart: Gm10 16 hpi CG
| Category | | | | |
|---|---|---|---|---|
### Chart: Gm11 16 hpi CG
| Category | | | | |
|---|---|---|---|---|
### Chart: Gm12 16 hpi CG
| Category | | | | |
|---|---|---|---|---|Proportion of methylation
30 Mb
10
20
40 Mb
30
10
20
40
30
10
20
50 Mb
### Chart: Gm13 16 hpi CG
| Category | | | | |
|---|---|---|---|---|
### Chart: Gm14 16 hpi CG
| Category | | | | |
|---|---|---|---|---|
### Chart: Gm15 16 hpi CG
| Category | | | | |
|---|---|---|---|---|40
30
10
20
50 Mb
40 Mb
30
10
20
40 Mb
30
10
20
### Chart: Gm18 16 hpi CG
| Category | | | | |
|---|---|---|---|---|
### Chart: Gm16 16 hpi CG
| Category | | | | |
|---|---|---|---|---|
### Chart: Gm17 16 hpi CG
| Category | | | | |
|---|---|---|---|---|40
30
10
20
50 Mb
40 Mb
30
10
20
30 Mb
10
20
### Chart: Gm20 16 hpi CG
| Category | | | | |
|---|---|---|---|---|
### Chart: Gm19 16 hpi CG
| Category | | | | |
|---|---|---|---|---|Colfax control
Colfax inoculated
Williams 82 control
Williams 82 inoculated
40
30
10
20
50 Mb
40 Mb
30
10
20
Supplementary Fig. 3. CG methylation distribution across the 20 soybean chromosomes at 16 hpi.

## Slide 4
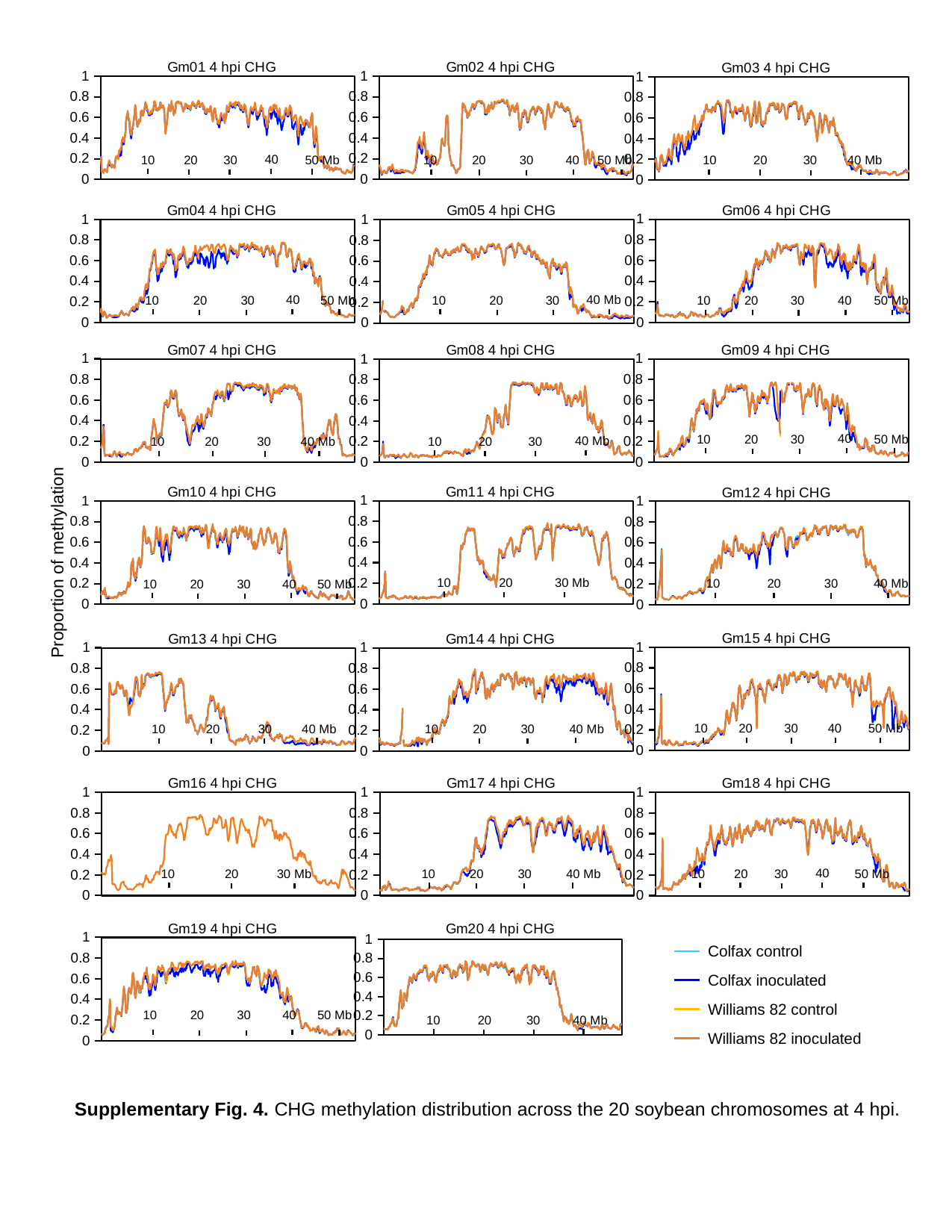

### Chart: Gm01 4 hpi CHG
| Category | cf_4_CHG_c | cf_4_CHG_t | wm_4_CHG_c | wm_4_CHG_t |
|---|---|---|---|---|
### Chart: Gm02 4 hpi CHG
| Category | | | | |
|---|---|---|---|---|
### Chart: Gm03 4 hpi CHG
| Category | | | | |
|---|---|---|---|---|40
30
10
20
50 Mb
40
30
10
20
50 Mb
40 Mb
30
10
20
### Chart: Gm06 4 hpi CHG
| Category | | | | |
|---|---|---|---|---|
### Chart: Gm04 4 hpi CHG
| Category | | | | |
|---|---|---|---|---|
### Chart: Gm05 4 hpi CHG
| Category | | | | |
|---|---|---|---|---|40
30
10
20
50 Mb
40 Mb
30
10
20
40
30
10
20
50 Mb
### Chart: Gm07 4 hpi CHG
| Category | | | | |
|---|---|---|---|---|
### Chart: Gm09 4 hpi CHG
| Category | | | | |
|---|---|---|---|---|
### Chart: Gm08 4 hpi CHG
| Category | | | | |
|---|---|---|---|---|40
30
10
20
50 Mb
40 Mb
30
10
20
40 Mb
30
10
20
### Chart: Gm11 4 hpi CHG
| Category | | | | |
|---|---|---|---|---|
### Chart: Gm10 4 hpi CHG
| Category | | | | |
|---|---|---|---|---|
### Chart: Gm12 4 hpi CHG
| Category | | | | |
|---|---|---|---|---|Proportion of methylation
30 Mb
10
20
40 Mb
30
10
20
40
30
10
20
50 Mb
### Chart: Gm15 4 hpi CHG
| Category | | | | |
|---|---|---|---|---|
### Chart: Gm13 4 hpi CHG
| Category | | | | |
|---|---|---|---|---|
### Chart: Gm14 4 hpi CHG
| Category | | | | |
|---|---|---|---|---|40
30
10
20
50 Mb
40 Mb
30
10
20
40 Mb
30
10
20
### Chart: Gm16 4 hpi CHG
| Category | | | | |
|---|---|---|---|---|
### Chart: Gm17 4 hpi CHG
| Category | | | | |
|---|---|---|---|---|
### Chart: Gm18 4 hpi CHG
| Category | | | | |
|---|---|---|---|---|40
30
10
20
50 Mb
40 Mb
30
10
20
30 Mb
10
20
### Chart: Gm19 4 hpi CHG
| Category | | | | |
|---|---|---|---|---|
### Chart: Gm20 4 hpi CHG
| Category | | | | |
|---|---|---|---|---|Colfax control
Colfax inoculated
Williams 82 control
Williams 82 inoculated
40
30
10
20
50 Mb
40 Mb
30
10
20
Supplementary Fig. 4. CHG methylation distribution across the 20 soybean chromosomes at 4 hpi.

## Slide 5
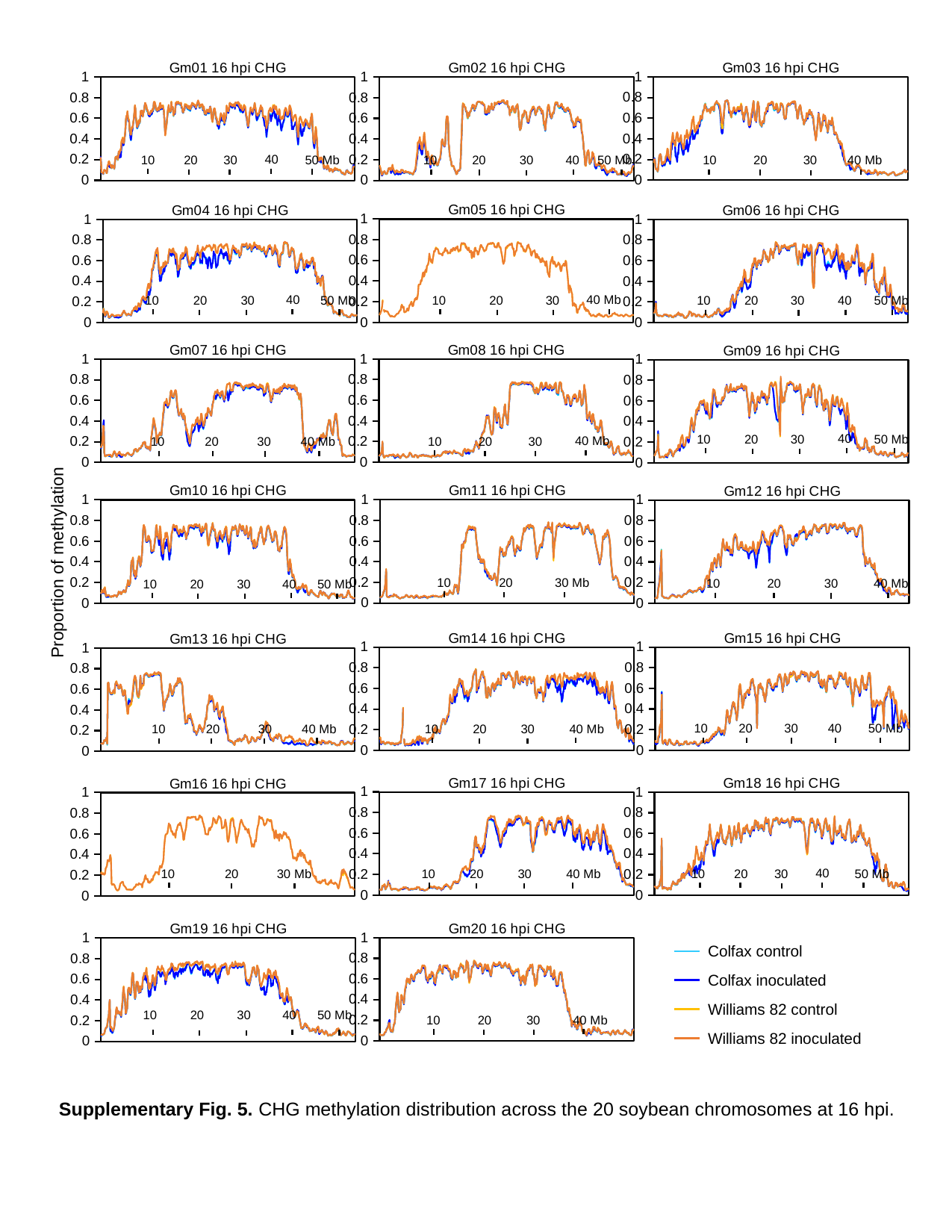

### Chart: Gm03 16 hpi CHG
| Category | | | | |
|---|---|---|---|---|
### Chart: Gm01 16 hpi CHG
| Category | cf_16_CHG_c | cf_16_CHG_t | wm_16_CHG_c | wm_16_CHG_t |
|---|---|---|---|---|
### Chart: Gm02 16 hpi CHG
| Category | | | | |
|---|---|---|---|---|40
30
10
20
50 Mb
40
30
10
20
50 Mb
40 Mb
30
10
20
### Chart: Gm05 16 hpi CHG
| Category | | | | |
|---|---|---|---|---|
### Chart: Gm04 16 hpi CHG
| Category | | | | |
|---|---|---|---|---|
### Chart: Gm06 16 hpi CHG
| Category | | | | |
|---|---|---|---|---|40
30
10
20
50 Mb
40 Mb
30
10
20
40
30
10
20
50 Mb
### Chart: Gm08 16 hpi CHG
| Category | | | | |
|---|---|---|---|---|
### Chart: Gm07 16 hpi CHG
| Category | | | | |
|---|---|---|---|---|
### Chart: Gm09 16 hpi CHG
| Category | | | | |
|---|---|---|---|---|40
30
10
20
50 Mb
40 Mb
30
10
20
40 Mb
30
10
20
### Chart: Gm11 16 hpi CHG
| Category | | | | |
|---|---|---|---|---|
### Chart: Gm10 16 hpi CHG
| Category | | | | |
|---|---|---|---|---|
### Chart: Gm12 16 hpi CHG
| Category | | | | |
|---|---|---|---|---|Proportion of methylation
30 Mb
10
20
40 Mb
30
10
20
40
30
10
20
50 Mb
### Chart: Gm15 16 hpi CHG
| Category | | | | |
|---|---|---|---|---|
### Chart: Gm14 16 hpi CHG
| Category | | | | |
|---|---|---|---|---|
### Chart: Gm13 16 hpi CHG
| Category | | | | |
|---|---|---|---|---|40
30
10
20
50 Mb
40 Mb
30
10
20
40 Mb
30
10
20
### Chart: Gm17 16 hpi CHG
| Category | | | | |
|---|---|---|---|---|
### Chart: Gm18 16 hpi CHG
| Category | | | | |
|---|---|---|---|---|
### Chart: Gm16 16 hpi CHG
| Category | | | | |
|---|---|---|---|---|40
30
10
20
50 Mb
40 Mb
30
10
20
30 Mb
10
20
### Chart: Gm20 16 hpi CHG
| Category | | | | |
|---|---|---|---|---|
### Chart: Gm19 16 hpi CHG
| Category | | | | |
|---|---|---|---|---|Colfax control
Colfax inoculated
Williams 82 control
Williams 82 inoculated
40
30
10
20
50 Mb
40 Mb
30
10
20
Supplementary Fig. 5. CHG methylation distribution across the 20 soybean chromosomes at 16 hpi.

## Slide 6
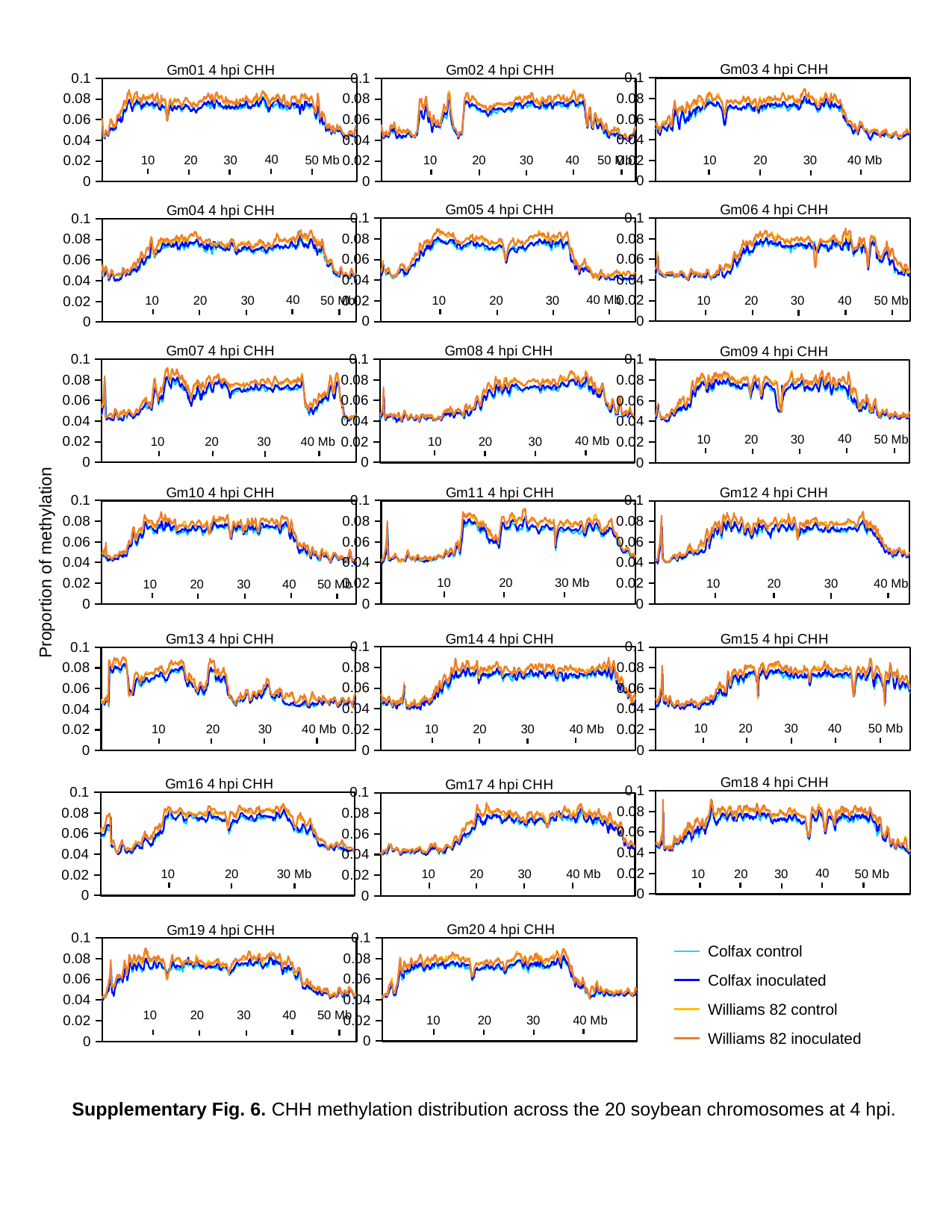

### Chart: Gm03 4 hpi CHH
| Category | | | | |
|---|---|---|---|---|
### Chart: Gm01 4 hpi CHH
| Category | cf_4_CHH_c | cf_4_CHH_t | wm_4_CHH_c | wm_4_CHH_t |
|---|---|---|---|---|
### Chart: Gm02 4 hpi CHH
| Category | | | | |
|---|---|---|---|---|40
30
10
20
50 Mb
40
30
10
20
50 Mb
40 Mb
30
10
20
### Chart: Gm06 4 hpi CHH
| Category | | | | |
|---|---|---|---|---|
### Chart: Gm05 4 hpi CHH
| Category | | | | |
|---|---|---|---|---|
### Chart: Gm04 4 hpi CHH
| Category | | | | |
|---|---|---|---|---|40
30
10
20
50 Mb
40 Mb
30
10
20
40
30
10
20
50 Mb
### Chart: Gm07 4 hpi CHH
| Category | | | | |
|---|---|---|---|---|
### Chart: Gm08 4 hpi CHH
| Category | | | | |
|---|---|---|---|---|
### Chart: Gm09 4 hpi CHH
| Category | | | | |
|---|---|---|---|---|40
30
10
20
50 Mb
40 Mb
30
10
20
40 Mb
30
10
20
### Chart: Gm10 4 hpi CHH
| Category | | | | |
|---|---|---|---|---|
### Chart: Gm11 4 hpi CHH
| Category | | | | |
|---|---|---|---|---|
### Chart: Gm12 4 hpi CHH
| Category | | | | |
|---|---|---|---|---|Proportion of methylation
30 Mb
10
20
40 Mb
30
10
20
40
30
10
20
50 Mb
### Chart: Gm14 4 hpi CHH
| Category | | | | |
|---|---|---|---|---|
### Chart: Gm15 4 hpi CHH
| Category | | | | |
|---|---|---|---|---|
### Chart: Gm13 4 hpi CHH
| Category | | | | |
|---|---|---|---|---|40
30
10
20
50 Mb
40 Mb
30
10
20
40 Mb
30
10
20
### Chart: Gm18 4 hpi CHH
| Category | | | | |
|---|---|---|---|---|
### Chart: Gm16 4 hpi CHH
| Category | | | | |
|---|---|---|---|---|
### Chart: Gm17 4 hpi CHH
| Category | | | | |
|---|---|---|---|---|40
30
10
20
50 Mb
40 Mb
30
10
20
30 Mb
10
20
### Chart: Gm20 4 hpi CHH
| Category | | | | |
|---|---|---|---|---|
### Chart: Gm19 4 hpi CHH
| Category | | | | |
|---|---|---|---|---|Colfax control
Colfax inoculated
Williams 82 control
Williams 82 inoculated
40
30
10
20
50 Mb
40 Mb
30
10
20
Supplementary Fig. 6. CHH methylation distribution across the 20 soybean chromosomes at 4 hpi.

## Slide 7
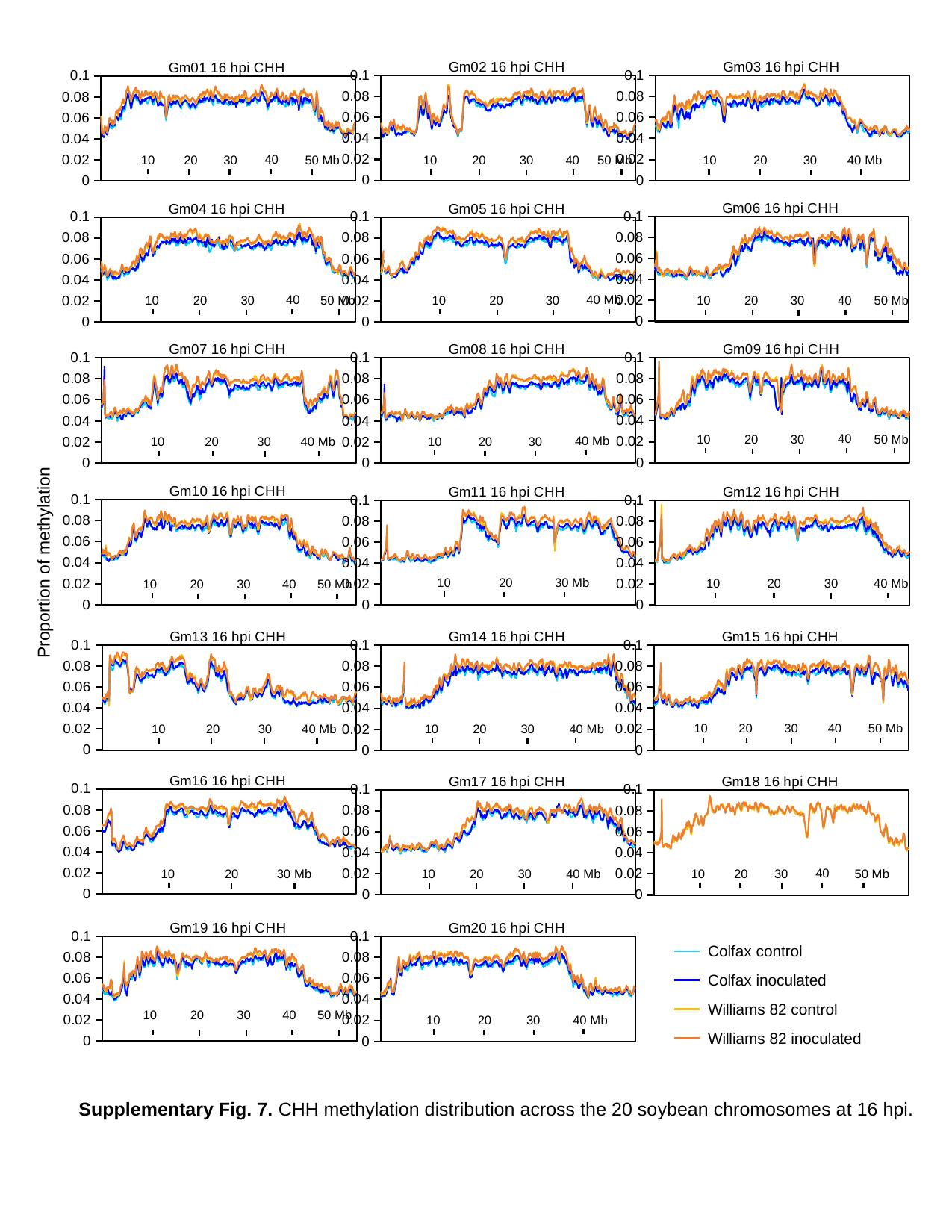

### Chart: Gm02 16 hpi CHH
| Category | | | | |
|---|---|---|---|---|
### Chart: Gm03 16 hpi CHH
| Category | | | | |
|---|---|---|---|---|
### Chart: Gm01 16 hpi CHH
| Category | cf_16_CHH_c | cf_16_CHH_t | wm_16_CHH_ | wm_16_CHH_t |
|---|---|---|---|---|40
30
10
20
50 Mb
40
30
10
20
50 Mb
40 Mb
30
10
20
### Chart: Gm06 16 hpi CHH
| Category | | | | |
|---|---|---|---|---|
### Chart: Gm04 16 hpi CHH
| Category | | | | |
|---|---|---|---|---|
### Chart: Gm05 16 hpi CHH
| Category | | | | |
|---|---|---|---|---|40
30
10
20
50 Mb
40 Mb
30
10
20
40
30
10
20
50 Mb
### Chart: Gm09 16 hpi CHH
| Category | | | | |
|---|---|---|---|---|
### Chart: Gm08 16 hpi CHH
| Category | | | | |
|---|---|---|---|---|
### Chart: Gm07 16 hpi CHH
| Category | | | | |
|---|---|---|---|---|40
30
10
20
50 Mb
40 Mb
30
10
20
40 Mb
30
10
20
### Chart: Gm10 16 hpi CHH
| Category | | | | |
|---|---|---|---|---|
### Chart: Gm11 16 hpi CHH
| Category | | | | |
|---|---|---|---|---|
### Chart: Gm12 16 hpi CHH
| Category | | | | |
|---|---|---|---|---|Proportion of methylation
30 Mb
10
20
40 Mb
30
10
20
40
30
10
20
50 Mb
### Chart: Gm13 16 hpi CHH
| Category | | | | |
|---|---|---|---|---|
### Chart: Gm15 16 hpi CHH
| Category | | | | |
|---|---|---|---|---|
### Chart: Gm14 16 hpi CHH
| Category | | | | |
|---|---|---|---|---|40
30
10
20
50 Mb
40 Mb
30
10
20
40 Mb
30
10
20
### Chart: Gm16 16 hpi CHH
| Category | | | | |
|---|---|---|---|---|
### Chart: Gm17 16 hpi CHH
| Category | | | | |
|---|---|---|---|---|
### Chart: Gm18 16 hpi CHH
| Category | | | | |
|---|---|---|---|---|40
30
10
20
50 Mb
40 Mb
30
10
20
30 Mb
10
20
### Chart: Gm19 16 hpi CHH
| Category | | | | |
|---|---|---|---|---|
### Chart: Gm20 16 hpi CHH
| Category | | | | |
|---|---|---|---|---|Colfax control
Colfax inoculated
Williams 82 control
Williams 82 inoculated
40
30
10
20
50 Mb
40 Mb
30
10
20
Supplementary Fig. 7. CHH methylation distribution across the 20 soybean chromosomes at 16 hpi.

## Slide 8
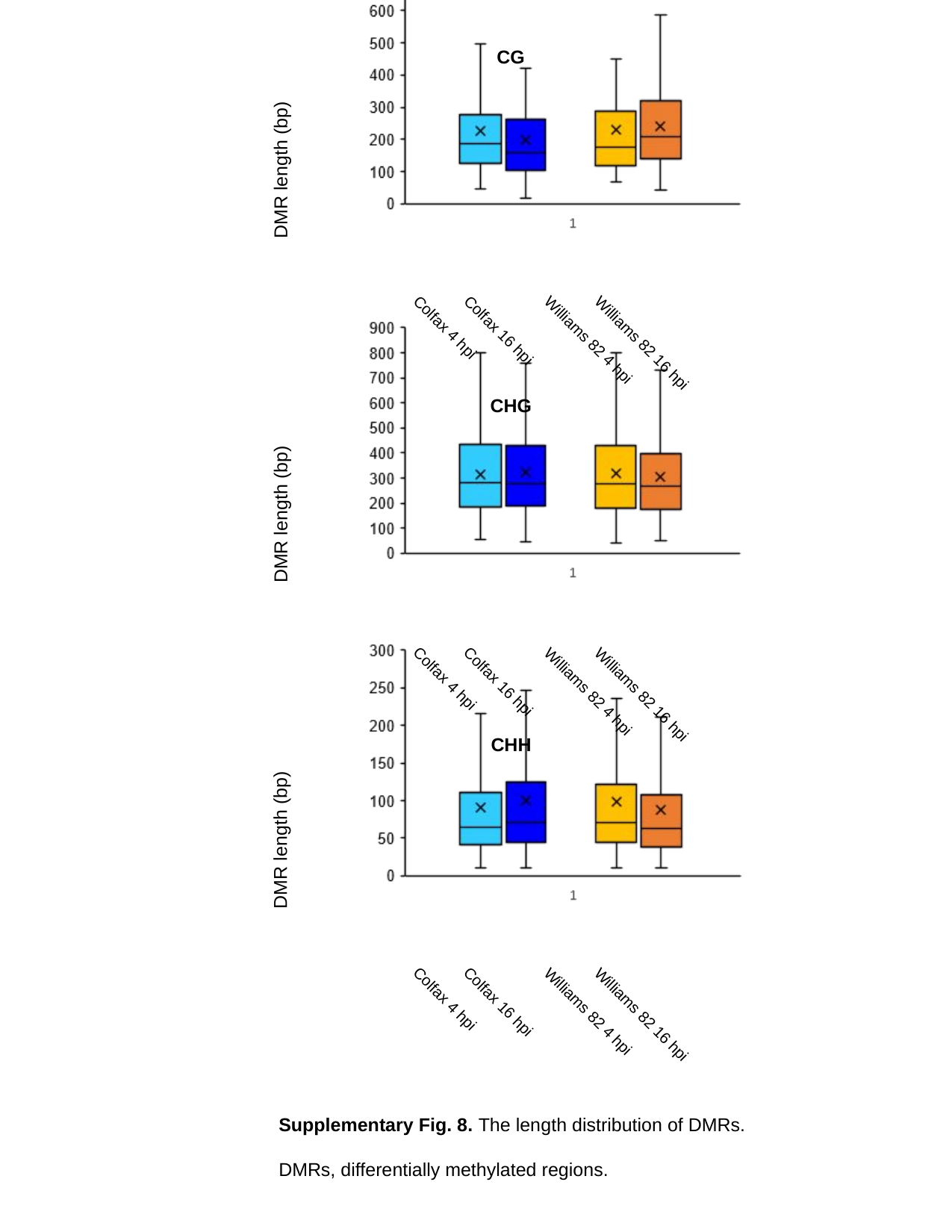

CG
DMR length (bp)
Colfax 16 hpi
Colfax 4 hpi
Williams 82 4 hpi
Williams 82 16 hpi
CHG
DMR length (bp)
Colfax 16 hpi
Colfax 4 hpi
Williams 82 4 hpi
Williams 82 16 hpi
CHH
DMR length (bp)
Colfax 16 hpi
Colfax 4 hpi
Williams 82 4 hpi
Williams 82 16 hpi
Supplementary Fig. 8. The length distribution of DMRs.
DMRs, differentially methylated regions.

## Slide 9
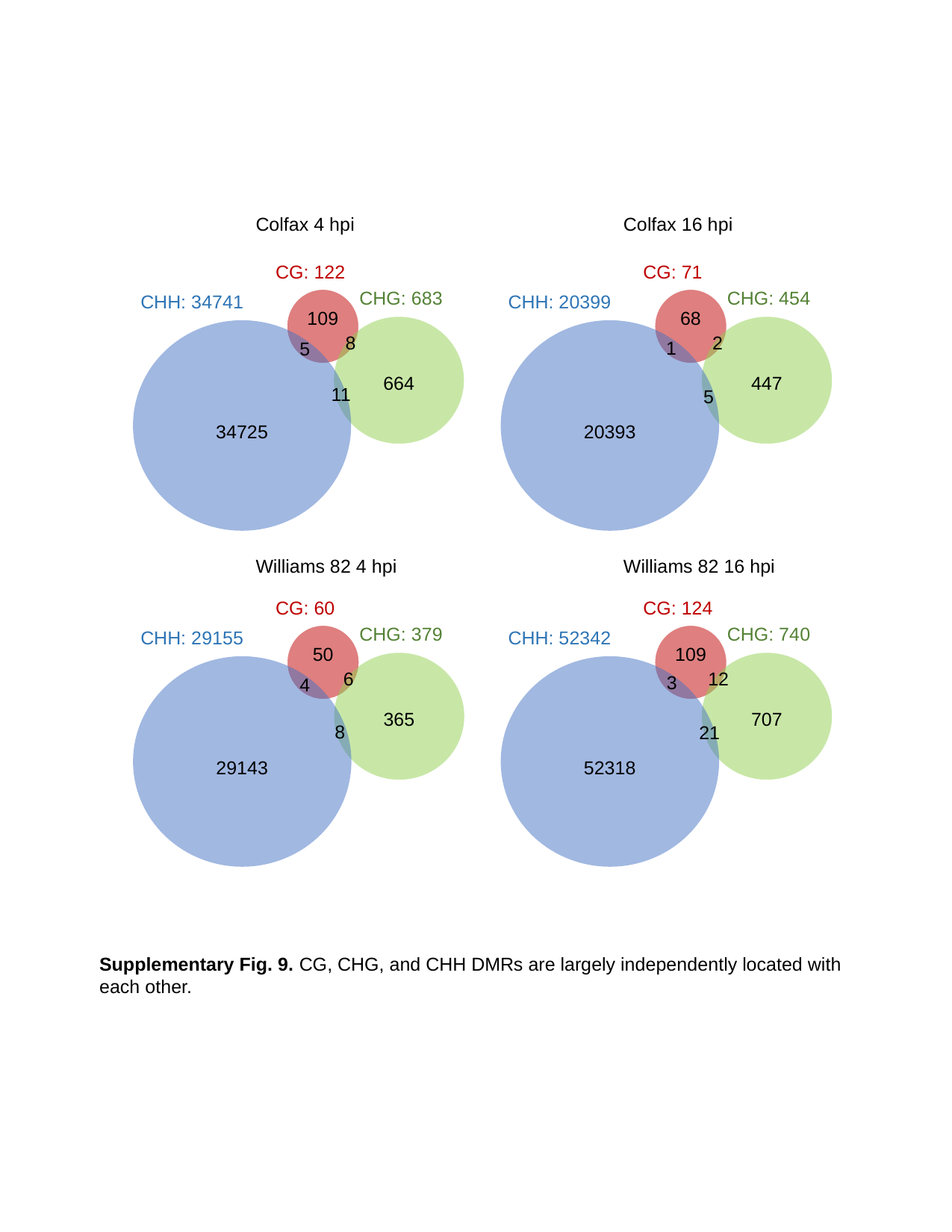

Colfax 4 hpi
Colfax 16 hpi
2
1
5
CG: 71
CHG: 454
CHH: 20399
8
5
11
CG: 122
CHG: 683
CHH: 34741
Williams 82 16 hpi
12
3
21
CG: 124
CHG: 740
CHH: 52342
Williams 82 4 hpi
6
4
8
CG: 60
CHG: 379
CHH: 29155
Supplementary Fig. 9. CG, CHG, and CHH DMRs are largely independently located with each other.

## Slide 10
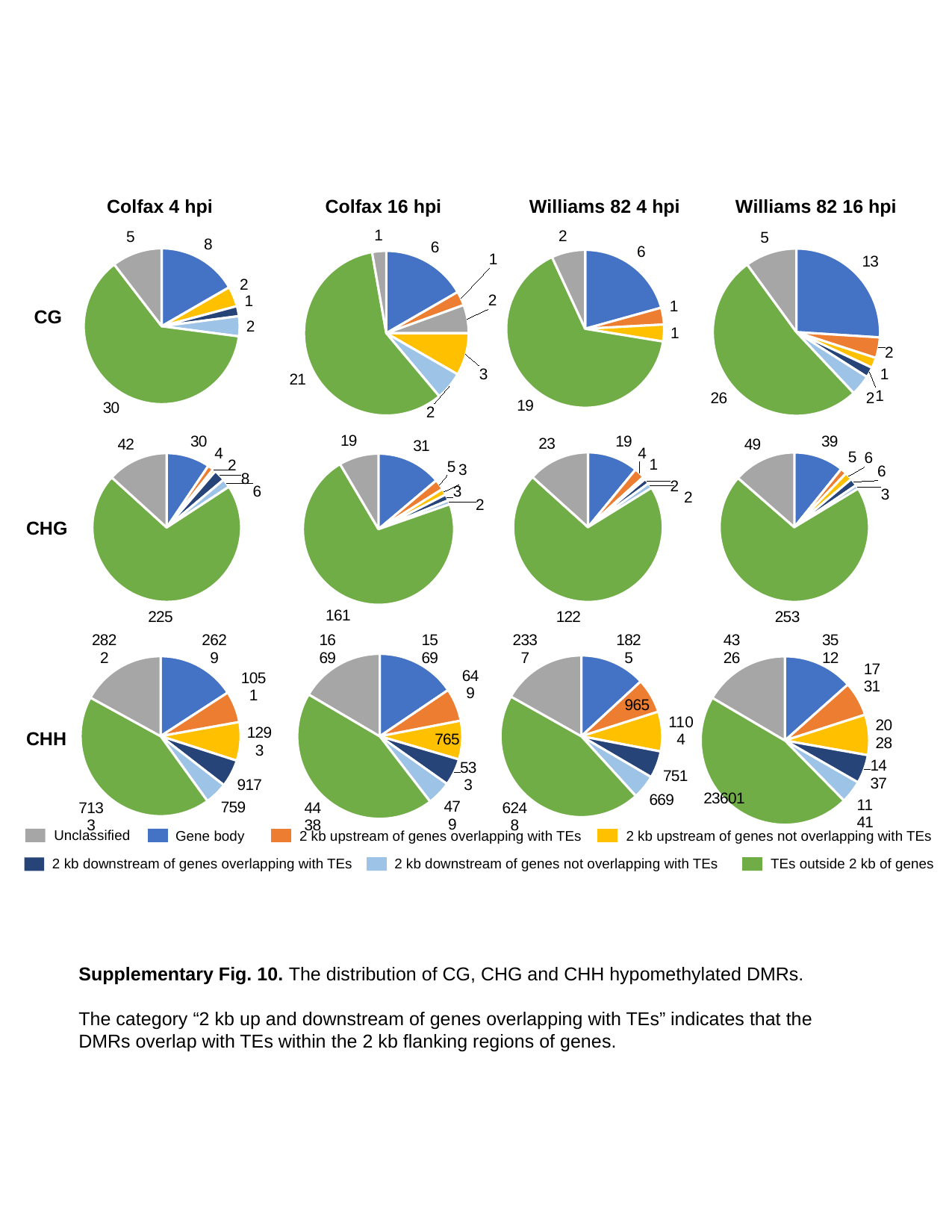

Colfax 4 hpi
Williams 82 16 hpi
Colfax 16 hpi
Williams 82 4 hpi
### Chart
| Category | Column1 |
|---|---|
| genebody | 8.0 |
| 2 kb upstream of gene overlapping TEs | 0.0 |
| 2 kb upstream of gene not overlapping TEs | 2.0 |
| 2 kb downstream of genes overlapping TEs | 1.0 |
| 2 kb downstream of genes not overlapping TEs | 2.0 |
| TEs | 30.0 |
| unclassified | 5.0 |
### Chart
| Category | Column1 |
|---|---|
| genebody | 6.0 |
| 2 kb upstream of gene overlapping TEs | 1.0 |
| 2 kb upstream of gene not overlapping TEs | 2.0 |
| 2 kb downstream of genes overlapping TEs | 3.0 |
| 2 kb downstream of genes not overlapping TEs | 2.0 |
| TEs | 21.0 |
| unclassified | 1.0 |
### Chart
| Category | Column1 |
|---|---|
| genebody | 6.0 |
| 2 kb upstream of gene overlapping TEs | 1.0 |
| 2 kb upstream of gene not overlapping TEs | 0.0 |
| 2 kb downstream of genes overlapping TEs | 1.0 |
| 2 kb downstream of genes not overlapping TEs | 0.0 |
| TEs | 19.0 |
| unclassified | 2.0 |
### Chart
| Category | Column1 |
|---|---|
| genebody | 13.0 |
| 2 kb upstream of gene overlapping TEs | 2.0 |
| 2 kb upstream of gene not overlapping TEs | 1.0 |
| 2 kb downstream of genes overlapping TEs | 1.0 |
| 2 kb downstream of genes not overlapping TEs | 2.0 |
| TEs | 26.0 |
| unclassified | 5.0 |CG
### Chart
| Category |
|---|
### Chart
| Category | Column1 |
|---|---|
| genebody | 30.0 |
| 2 kb upstream of gene overlapping TEs | 4.0 |
| 2 kb upstream of gene not overlapping TEs | 2.0 |
| 2 kb downstream of genes overlapping TEs | 8.0 |
| 2 kb downstream of genes not overlapping TEs | 6.0 |
| TEs | 225.0 |
| unclassified | 42.0 |
### Chart
| Category | Column1 |
|---|---|
| genebody | 31.0 |
| 2 kb upstream of gene overlapping TEs | 5.0 |
| 2 kb upstream of gene not overlapping TEs | 3.0 |
| 2 kb downstream of genes overlapping TEs | 3.0 |
| 2 kb downstream of genes not overlapping TEs | 2.0 |
| TEs | 161.0 |
| unclassified | 19.0 |
### Chart
| Category | Column1 |
|---|---|
| genebody | 19.0 |
| 2 kb upstream of gene overlapping TEs | 4.0 |
| 2 kb upstream of gene not overlapping TEs | 1.0 |
| 2 kb downstream of genes overlapping TEs | 2.0 |
| 2 kb downstream of genes not overlapping TEs | 2.0 |
| TEs | 122.0 |
| unclassified | 23.0 |
### Chart
| Category | Column1 |
|---|---|
| genebody | 39.0 |
| 2 kb upstream of gene overlapping TEs | 5.0 |
| 2 kb upstream of gene not overlapping TEs | 6.0 |
| 2 kb downstream of genes overlapping TEs | 6.0 |
| 2 kb downstream of genes not overlapping TEs | 3.0 |
| TEs | 253.0 |
| unclassified | 49.0 |CHG
### Chart
| Category | Column1 |
|---|---|
| genebody | 2629.0 |
| 2 kb upstream of gene overlapping TEs | 1051.0 |
| 2 kb upstream of gene not overlapping TEs | 1293.0 |
| 2 kb downstream of genes overlapping TEs | 917.0 |
| 2 kb downstream of genes not overlapping TEs | 759.0 |
| TEs | 7133.0 |
| unclassified | 2822.0 |
### Chart
| Category | Column1 |
|---|---|
| genebody | 1569.0 |
| 2 kb upstream of gene overlapping TEs | 649.0 |
| 2 kb upstream of gene not overlapping TEs | 765.0 |
| 2 kb downstream of genes overlapping TEs | 533.0 |
| 2 kb downstream of genes not overlapping TEs | 479.0 |
| TEs | 4438.0 |
| unclassified | 1669.0 |
### Chart
| Category | Column1 |
|---|---|
| genebody | 1825.0 |
| 2 kb upstream of gene overlapping TEs | 965.0 |
| 2 kb upstream of gene not overlapping TEs | 1104.0 |
| 2 kb downstream of genes overlapping TEs | 751.0 |
| 2 kb downstream of genes not overlapping TEs | 669.0 |
| TEs | 6248.0 |
| unclassified | 2337.0 |
### Chart
| Category | Column1 |
|---|---|
| genebody | 3512.0 |
| 2 kb upstream of gene overlapping TEs | 1731.0 |
| 2 kb upstream of gene not overlapping TEs | 2028.0 |
| 2 kb downstream of genes overlapping TEs | 1437.0 |
| 2 kb downstream of genes not overlapping TEs | 1141.0 |
| TEs | 11989.0 |
| unclassified | 4326.0 |CHH
Unclassified
Gene body
2 kb upstream of genes overlapping with TEs
2 kb upstream of genes not overlapping with TEs
2 kb downstream of genes overlapping with TEs
2 kb downstream of genes not overlapping with TEs
TEs outside 2 kb of genes
Supplementary Fig. 10. The distribution of CG, CHG and CHH hypomethylated DMRs.
The category “2 kb up and downstream of genes overlapping with TEs” indicates that the DMRs overlap with TEs within the 2 kb flanking regions of genes.

## Slide 11
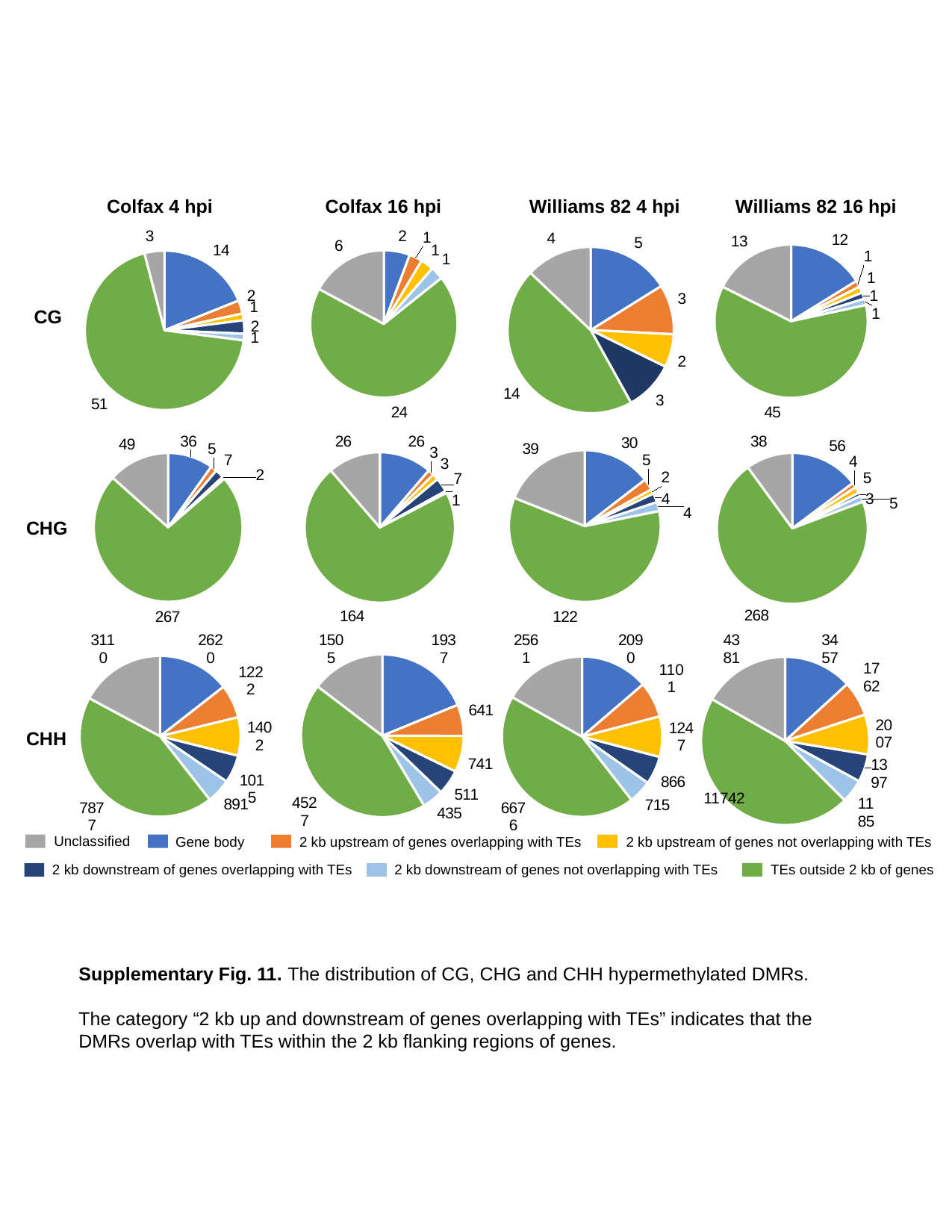

Colfax 4 hpi
Williams 82 16 hpi
Colfax 16 hpi
Williams 82 4 hpi
### Chart
| Category | Column1 |
|---|---|
| genebody | 14.0 |
| 2 kb upstream of gene overlapping TEs | 2.0 |
| 2 kb upstream of gene not overlapping TEs | 1.0 |
| 2 kb downstream of genes overlapping TEs | 2.0 |
| 2 kb downstream of genes not overlapping TEs | 1.0 |
| TEs | 51.0 |
| unclassified | 3.0 |
### Chart
| Category | Column1 |
|---|---|
| genebody | 2.0 |
| 2 kb upstream of gene overlapping TEs | 1.0 |
| 2 kb upstream of gene not overlapping TEs | 1.0 |
| 2 kb downstream of genes overlapping TEs | 0.0 |
| 2 kb downstream of genes not overlapping TEs | 1.0 |
| TEs | 24.0 |
| unclassified | 6.0 |
### Chart
| Category | Column1 |
|---|---|
| genebody | 5.0 |
| 2 kb upstream of gene overlapping TEs | 3.0 |
| 2 kb upstream of gene not overlapping TEs | 2.0 |
| 2 kb downstream of genes overlapping TEs | 3.0 |
| 2 kb downstream of genes not overlapping TEs | 0.0 |
| TEs | 14.0 |
| unclassified | 4.0 |
### Chart
| Category | Column1 |
|---|---|
| genebody | 12.0 |
| 2 kb upstream of gene overlapping TEs | 1.0 |
| 2 kb upstream of gene not overlapping TEs | 1.0 |
| 2 kb downstream of genes overlapping TEs | 1.0 |
| 2 kb downstream of genes not overlapping TEs | 1.0 |
| TEs | 45.0 |
| unclassified | 13.0 |CG
### Chart
| Category | Column1 |
|---|---|
| genebody | 36.0 |
| 2 kb upstream of gene overlapping TEs | 5.0 |
| 2 kb upstream of gene not overlapping TEs | 0.0 |
| 2 kb downstream of genes overlapping TEs | 7.0 |
| 2 kb downstream of genes not overlapping TEs | 2.0 |
| TEs | 267.0 |
| unclassified | 49.0 |
### Chart
| Category | Column1 |
|---|---|
| genebody | 26.0 |
| 2 kb upstream of gene overlapping TEs | 3.0 |
| 2 kb upstream of gene not overlapping TEs | 3.0 |
| 2 kb downstream of genes overlapping TEs | 7.0 |
| 2 kb downstream of genes not overlapping TEs | 1.0 |
| TEs | 164.0 |
| unclassified | 26.0 |
### Chart
| Category | Column1 |
|---|---|
| genebody | 30.0 |
| 2 kb upstream of gene overlapping TEs | 5.0 |
| 2 kb upstream of gene not overlapping TEs | 2.0 |
| 2 kb downstream of genes overlapping TEs | 4.0 |
| 2 kb downstream of genes not overlapping TEs | 4.0 |
| TEs | 122.0 |
| unclassified | 39.0 |
### Chart
| Category | Column1 |
|---|---|
| genebody | 56.0 |
| 2 kb upstream of gene overlapping TEs | 4.0 |
| 2 kb upstream of gene not overlapping TEs | 5.0 |
| 2 kb downstream of genes overlapping TEs | 3.0 |
| 2 kb downstream of genes not overlapping TEs | 5.0 |
| TEs | 268.0 |
| unclassified | 38.0 |CHG
### Chart
| Category | Column1 |
|---|---|
| genebody | 2620.0 |
| 2 kb upstream of gene overlapping TEs | 1222.0 |
| 2 kb upstream of gene not overlapping TEs | 1402.0 |
| 2 kb downstream of genes overlapping TEs | 1015.0 |
| 2 kb downstream of genes not overlapping TEs | 891.0 |
| TEs | 7877.0 |
| unclassified | 3110.0 |
### Chart
| Category | Column1 |
|---|---|
| genebody | 1937.0 |
| 2 kb upstream of gene overlapping TEs | 641.0 |
| 2 kb upstream of gene not overlapping TEs | 741.0 |
| 2 kb downstream of genes overlapping TEs | 511.0 |
| 2 kb downstream of genes not overlapping TEs | 435.0 |
| TEs | 4527.0 |
| unclassified | 1505.0 |
### Chart
| Category | Column1 |
|---|---|
| genebody | 2090.0 |
| 2 kb upstream of gene overlapping TEs | 1101.0 |
| 2 kb upstream of gene not overlapping TEs | 1247.0 |
| 2 kb downstream of genes overlapping TEs | 866.0 |
| 2 kb downstream of genes not overlapping TEs | 715.0 |
| TEs | 6676.0 |
| unclassified | 2561.0 |
### Chart
| Category | Column1 |
|---|---|
| genebody | 3457.0 |
| 2 kb upstream of gene overlapping TEs | 1762.0 |
| 2 kb upstream of gene not overlapping TEs | 2007.0 |
| 2 kb downstream of genes overlapping TEs | 1397.0 |
| 2 kb downstream of genes not overlapping TEs | 1185.0 |
| TEs | 11989.0 |
| unclassified | 4381.0 |CHH
Unclassified
Gene body
2 kb upstream of genes overlapping with TEs
2 kb upstream of genes not overlapping with TEs
2 kb downstream of genes overlapping with TEs
2 kb downstream of genes not overlapping with TEs
TEs outside 2 kb of genes
Supplementary Fig. 11. The distribution of CG, CHG and CHH hypermethylated DMRs.
The category “2 kb up and downstream of genes overlapping with TEs” indicates that the DMRs overlap with TEs within the 2 kb flanking regions of genes.

## Slide 12
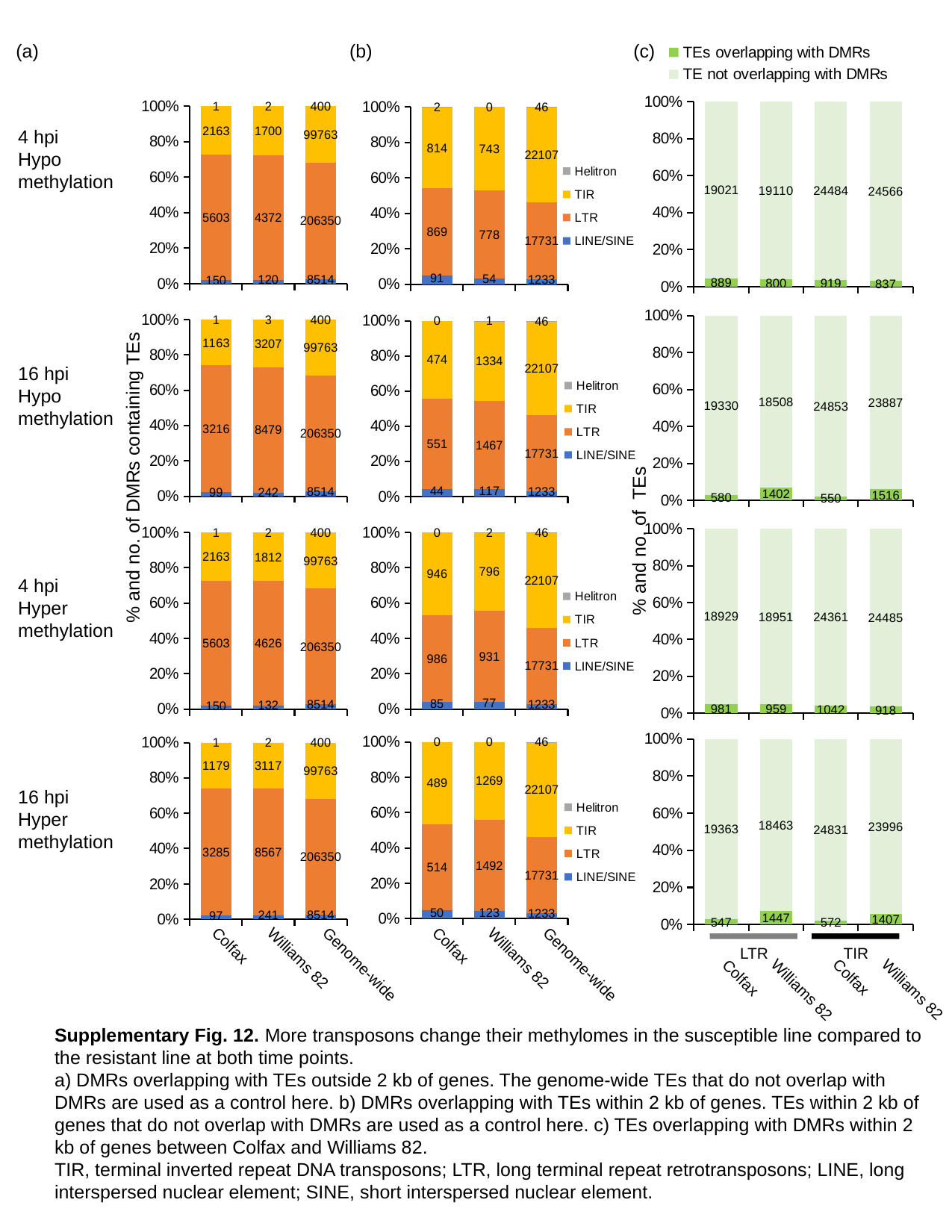

### Chart
| Category | TEs overlapping with DMRs | TE not overlapping with DMRs |
|---|---|---|
| colfax_LTR | 889.0 | 19021.0 |
| willams82_LTR | 800.0 | 19110.0 |
| colfax_TIR | 919.0 | 24484.0 |
| willams82_TIR | 837.0 | 24566.0 |(b)
(c)
(a)
### Chart
| Category | LINE/SINE | LTR | TIR | Helitron |
|---|---|---|---|---|
| colfax | 150.0 | 5603.0 | 2163.0 | 1.0 |
| willams82 | 120.0 | 4372.0 | 1700.0 | 2.0 |
| Genome | 8514.0 | 206350.0 | 99763.0 | 400.0 |
### Chart
| Category | LINE/SINE | LTR | TIR | Helitron |
|---|---|---|---|---|
| colfax | 91.0 | 869.0 | 814.0 | 2.0 |
| willams82 | 54.0 | 778.0 | 743.0 | 0.0 |
| Genome | 1233.0 | 17731.0 | 22107.0 | 46.0 |4 hpi
Hypo
methylation
### Chart
| Category | DMRs | Non-DMRs |
|---|---|---|
| colfax_LTR | 580.0 | 19330.0 |
| willams82_LTR | 1402.0 | 18508.0 |
| colfax_TIR | 550.0 | 24853.0 |
| willams82_TIR | 1516.0 | 23887.0 |
### Chart
| Category | LINE/SINE | LTR | TIR | Helitron |
|---|---|---|---|---|
| colfax | 99.0 | 3216.0 | 1163.0 | 1.0 |
| willams82 | 242.0 | 8479.0 | 3207.0 | 3.0 |
| Genome | 8514.0 | 206350.0 | 99763.0 | 400.0 |
### Chart
| Category | LINE/SINE | LTR | TIR | Helitron |
|---|---|---|---|---|
| colfax | 44.0 | 551.0 | 474.0 | 0.0 |
| willams82 | 117.0 | 1467.0 | 1334.0 | 1.0 |
| Genome | 1233.0 | 17731.0 | 22107.0 | 46.0 |16 hpi
Hypo
methylation
% and no. of DMRs containing TEs
### Chart
| Category | DMRs | Non-DMRs |
|---|---|---|
| colfax_LTR | 981.0 | 18929.0 |
| willams82_LTR | 959.0 | 18951.0 |
| colfax_TIR | 1042.0 | 24361.0 |
| willams82_TIR | 918.0 | 24485.0 |
### Chart
| Category | LINE/SINE | LTR | TIR | Helitron |
|---|---|---|---|---|
| colfax | 150.0 | 5603.0 | 2163.0 | 1.0 |
| willams82 | 132.0 | 4626.0 | 1812.0 | 2.0 |
| Genome | 8514.0 | 206350.0 | 99763.0 | 400.0 |
### Chart
| Category | LINE/SINE | LTR | TIR | Helitron |
|---|---|---|---|---|
| colfax | 85.0 | 986.0 | 946.0 | 0.0 |
| willams82 | 77.0 | 931.0 | 796.0 | 2.0 |
| Genome | 1233.0 | 17731.0 | 22107.0 | 46.0 |% and no. of TEs
4 hpi
Hyper
methylation
### Chart
| Category | DMRs | Non-DMRs |
|---|---|---|
| colfax_LTR | 547.0 | 19363.0 |
| willams82_LTR | 1447.0 | 18463.0 |
| colfax_TIR | 572.0 | 24831.0 |
| willams82_TIR | 1407.0 | 23996.0 |
### Chart
| Category | LINE/SINE | LTR | TIR | Helitron |
|---|---|---|---|---|
| colfax | 50.0 | 514.0 | 489.0 | 0.0 |
| willams82 | 123.0 | 1492.0 | 1269.0 | 0.0 |
| Genome | 1233.0 | 17731.0 | 22107.0 | 46.0 |
### Chart
| Category | LINE/SINE | LTR | TIR | Helitron |
|---|---|---|---|---|
| colfax | 97.0 | 3285.0 | 1179.0 | 1.0 |
| willams82 | 241.0 | 8567.0 | 3117.0 | 2.0 |
| Genome | 8514.0 | 206350.0 | 99763.0 | 400.0 |16 hpi
Hyper
methylation
Colfax
Williams 82
Genome-wide
Colfax
Williams 82
Genome-wide
LTR
TIR
Williams 82
Colfax
Williams 82
Colfax
Supplementary Fig. 12. More transposons change their methylomes in the susceptible line compared to the resistant line at both time points.
a) DMRs overlapping with TEs outside 2 kb of genes. The genome-wide TEs that do not overlap with DMRs are used as a control here. b) DMRs overlapping with TEs within 2 kb of genes. TEs within 2 kb of genes that do not overlap with DMRs are used as a control here. c) TEs overlapping with DMRs within 2 kb of genes between Colfax and Williams 82.
TIR, terminal inverted repeat DNA transposons; LTR, long terminal repeat retrotransposons; LINE, long interspersed nuclear element; SINE, short interspersed nuclear element.
